# *Synechococcus* nitrogen gene loss in iron-limited ocean regions

**DOI:** 10.1038/s43705-023-00314-9

**Published:** 2023-10-02

**Authors:** Garrett Sharpe, Liang Zhao, Meredith G. Meyer, Weida Gong, Shannon M. Burns, Allesandro Tagliabue, Kristen N. Buck, Alyson E. Santoro, Jason R. Graff, Adrian Marchetti, Scott Gifford

**Affiliations:** 1 Environment Ecology and Energy Program, University of North Carolina at Chapel Hill, Chapel Hill, NC USA; 2 Earth, Marine and Environmental Sciences, University of North Carolina at Chapel Hill, Chapel Hill, NC USA; 3 College of Marine Science, University of South Florida, St. Petersburg, FL USA; 4 School of Environmental Sciences, University of Liverpool, Liverpool, UK; 5 College of Earth, Ocean, and Atmospheric Sciences, Oregon State University, Corvallis, OR USA; 6 Department of Ecology, Evolution, and Marine Biology, University of California, Santa Barbara, CA USA; 7 Department of Botany and Plant Pathology, Oregon State University, Corvallis, OR USA

**Keywords:** Water microbiology, Biogeochemistry, Biogeochemistry, Microbial ecology

## Abstract

*Synechococcus* are the most abundant cyanobacteria in high latitude regions and are responsible for an estimated 17% of annual marine net primary productivity. Despite their biogeochemical importance, *Synechococcus* populations have been unevenly sampled across the ocean, with most studies focused on low-latitude strains. In particular, the near absence of *Synechococcus* genomes from high-latitude, High Nutrient Low Chlorophyll (HNLC) regions leaves a gap in our knowledge of picocyanobacterial adaptations to iron limitation and their influence on carbon, nitrogen, and iron cycles. We examined *Synechococcus* populations from the subarctic North Pacific, a well-characterized HNLC region, with quantitative metagenomics. Assembly with short and long reads produced two near complete *Synechococcus* metagenome-assembled genomes (MAGs). Quantitative metagenome-derived abundances of these populations matched well with flow cytometry counts, and the *Synechococcus* MAGs were estimated to comprise >99% of the *Synechococcus* at Station P. Whereas the Station P *Synechococcus* MAGs contained multiple genes for adaptation to iron limitation, both genomes lacked genes for uptake and assimilation of nitrate and nitrite, suggesting a dependence on ammonium, urea, and other forms of recycled nitrogen leading to reduced iron requirements. A global analysis of *Synechococcus* nitrate reductase abundance in the TARA Oceans dataset found nitrate assimilation genes are also lower in other HNLC regions. We propose that nitrate and nitrite assimilation gene loss in *Synechococcus* may represent an adaptation to severe iron limitation in high-latitude regions where ammonium availability is higher. Our findings have implications for models that quantify the contribution of cyanobacteria to primary production and subsequent carbon export.

## Introduction

*Prochlorococcus* and *Synechococcus* are critical components of marine biogeochemical cycles, generating ~25% of the ocean’s annual net primary production and contributing significantly to carbon export [1, 2]. *Prochlorococcus* is largely restricted to equatorial and subtropical latitudes, while *Synechococcus* dominates cooler waters in regions of equatorial upwelling and high latitudes [3]. Both groups exhibit high levels of strain diversification due to niche specialization arising from variations in environmental conditions (light, temperature, nitrogen, phosphorus, etc.) including iron availability [4–6].

Iron (Fe) is an essential micronutrient as it is a required cofactor in photosynthetic and respiratory electron transport chains [7, 8]. Photosystems I and II require 12 and 3 iron atoms per photosystem, respectively, and the light-harvesting phycobilisome proteins are synthesized by iron-containing enzymes [8]. Iron is also required in other key metabolic functions, including nitrate and nitrite assimilation, with nitrate and nitrite reductases requiring 4 and 5 iron atoms per enzyme, respectively [9–11]. In High Nutrient Low Chlorophyll (HNLC) regions these cellular iron demands in combination with low iron bioavailability lead to iron limitation of primary production. Three major ocean regions have been identified as HNLC zones: the Equatorial Pacific, the Southern Ocean, and the subarctic North Pacific; together they represent roughly 30% of the world’s oceans [12]. These regions are characterized by low phytoplankton biomass and consistently high concentrations of macronutrients in the mixed layer resulting from incomplete utilization due to severe iron limitation [13, 14]. Nitrate (NO_3_^−^) concentrations for example are in the tens of micromolar range [14].

*Synechococcus* and *Prochlorococcus* strains have evolved diverse adaptations to iron limitation. In non-HNLC regions, *Prochlorococcus* are enriched in iron-storing ferritin genes and iron uptake regulators that enables growth at approximately ten-fold lower iron concentrations and a more rapid response to iron-stress relief, and low-light adapted *Prochlorococcus* also often possess siderophore transport operons in order to scavenge for iron at the deep chlorophyll maximum where competition for iron is more intense [15, 16]. In *Synechococcus*, a study comparing the Atlantic coastal strain WH8020 to the oligotrophic ocean strain WH8102 found that coastal strain WH8020 possesses multiple iron storage, stress regulation, and response genes that are intricately regulated under the dynamic iron conditions of the coastal environment [17]. By contrast, the pelagic *Synechococcus* strain WH8102 that was isolated from the primarily nitrogen-limited Sargasso Sea oligotrophic gyre lacks many of these iron-response genes and exhibit a more limited iron regulatory response [17].

Cyanobacteria, however, are relatively under-sampled in high-latitude HNLC regions, resulting in a major gap in understanding nutrient acquisition and adaptation strategies in these large, biogeochemically important regions. In tropical and subtropical HNLC regions, *Prochlorococcus* and *Synechococcus* have adapted to iron-limiting conditions by substituting iron requiring genes such as iron-sulfur (Fe-S) containing proteins with non-iron containing functional homologues [18, 19]. Several *Synechococcus* strains from the CRD1 and CRD2 clades, two phylogenetically distinct lineages of *Synechococcus* found in the equatorial Pacific HNLC region, exhibit adaptations to iron limitation such as high ferritin gene copy numbers (CRD1), multiple types of iron transporters (CRD1), and siderophore uptake (CRD2) [19, 20]. Although no members of these clades have yet been isolated or sequenced from outside of the equatorial Pacific, 16S-23S internally transcribed spacer (ITS) marker gene analysis has shown that members of the CRD1 and CRD2 clades, are also present in high-latitude HNLC waters [20]. These HNLC regions are not well represented in current bacterial metagenomic datasets, and all currently sequenced isolates, metagenome-assembled genomes (MAGs), and single cell assembled genomes (SAGs) from these clades are derived from low-latitude HNLC zones [2, 20–23].

The absence of high-latitude HNLC *Synechococcus* genomes leaves a substantial gap in our current knowledge of picocyanobacterial iron adaptation strategies and their importance to biogeochemical cycling. Here, we used quantitative metagenomics and genome assembly to enumerate and characterize cyanobacteria populations at Station P (Ocean Station Papa) to identify the strategies *Synechococcus* have evolved to succeed in iron-limited HNLC zones and their impact on biogeochemical cycling and carbon export.

## Methods

Samples were collected at Station P in the North Pacific in August and September 2018 as part of the NASA Export Processes in the Ocean from RemoTe Sensing (EXPORTS) expedition [24]. Flow cytometric enumeration of *Synechococcus* was performed on a Becton Dickinson Influx Cell Sorter (BD-ICS) flow cytometer while at sea following previously published protocols [25]. For uptake incubations, seawater was collected with a trace metal clean rosette, aliquoted into acid washed bottles, and inoculated with NaH^13^CO_3_ isotope and Na^15^NO_3_ isotope at 10% of ambient dissolved inorganic carbon (DIC) and NO_3_^−^ concentrations and incubated for 24 hours. Samples were gravity filtered through pre-combusted GF/F filters, dried and stored until onshore analysis at the University of California Davis Stable Isotope Facility [26]. Ambient nutrient concentrations were collected as described in Siegel et al. 2021 [24].

For the metagenomes, seawater was prefiltered through a 5 µm polycarbonate membrane filter and cells collected on 0.2 µm polyethersulfone (PES) membrane filter. DNA for short read sequencing was extracted from the filters using a DNeasy Powerwater kit. Three internal genomic standards were added immediately before starting the extraction. Metagenomes were sequenced with HiSeq 4000 as 2 ×150 bp reads, and can be accessed under the NCBI Bioproject PRJNA78533 (Biosamples 24695624-24695646). Metagenome reads were annotated with a DIAMOND search against the NCBI Refseq protein database [27]. Metagenome assembled genomes (MAGs) were assembled with metaspades and then mapped for read coverage with Bowtie2 [28, 29]. Contigs were each binned by MetaBAT, MaxBin, and CONCOCT, and then consolidated using DAS Tool and CheckM [30–35]. *Synechococcus* MAG quality was increased by performing several additional assemblies including Oxford nanopore long reads and manual curation (See supplemental for detailed methods). DNA for long read sequencing was obtained by phenol chloroform extraction. These Whole Genome Shotgun projects have been deposited at DDBJ/ENA/GenBank under the accessions JAVBIW000000000 for *Synechococcus* sp. SP1 and JAVBIX000000000 for *Synechococcus* sp. SP2.

*Synechococcus* volumetric abundances were derived from internal standard normalized metagenomes by *recA* and internal standard genome recovery as described in Gifford et al. [36] and Satinsky et al. [37]. Metagenome *recA* genes were identified by a DIAMOND homology search against a custom RecA protein database, and the resulting *recA* read counts were converted to volumetric abundances using the internal standard’s recovery ratio and volume of seawater filtered.

To calculate MAG volumetric abundances, a coverage-based ratio was calculated for each of the three internal standard genomes by mapping metagenome reads to the internal standard reference genomes. The coverage-based recovery ratio is derived via the following:

1 $${R}_{{{{{\mathrm{cov}}}}}}=\frac{{S}_{{{{{\mathrm{cov}}}}}}}{{S}_{a}}$$

2 $${M}_{a}=\frac{{M}_{{{{{\mathrm{cov}}}}}}}{{R}_{{{{{\mathrm{cov}}}}}}}$$

1. R_cov_: coverage-based recovery ratio.
  S_cov_: mean depth of coverage of internal standard genomes by metagenomic reads.
  S_a_: molecules of internal standard genomes added to the sample
2. M_a_: molecules of any genome (MAG) in the sample.

M_cov_: mean depth of coverage of any genome (MAG) in the sequence library.

Metagenomic reads were mapped onto the internal standard genomes with bowtie2 and the mean depth of coverage was calculated by dividing the total number of bases mapped by the size of the genomes. The mean depth of coverage represents the number of internal standard genome recovered through sequencing, and this was divided by the number of genomes added to the samples to get at the recovery ratio. The number of MAGs recovered from the sequence library was retrieved by mapping reads onto the MAG and calculating mean depth of coverage. The *Synechococcus* MAG volumetric abundances were then determined by mapping metagenome reads onto the *Synechococcus* MAG, calculating the mean depth of coverage, and then dividing by the coverage-based recovery ratio and volume of seawater filtered.

For comparison of *Synechococcus* nitrogen gene frequencies between our metagenomes and TARA metagenomes, nitrogen gene (nitrate reductase, nitrite reductase, and ammonia transporter) to *recA* ratios were calculated using the TARA 0.2–3.0 µm fraction at 5 m for each TARA Station. The raw TARA metagenome reads were processed in the same manner as the Station P metagenome dataset. A diamond search against NarB, FocA, Amt, and RecA-specific NCBI refseq databases narrowed down the gene hits, and a following diamond BLAST of the resulting gene hits against the entire NCBI refseq v95 databased eliminated false positive hits. For *recA*, an additional diamond search against the GTDB RecA database was used to obtain GTDB taxonomic identifications. *Synechococcus* nitrogen and recombinase A genes were pulled out of the total hits, and hits with *Synechococcus* annotations in both NCBI and GTDB were quantified and used to calculate the nitrogen gene to *recA* ratios. Detailed descriptions of sample collection, processing, and all bioinformatic analysis can be found in the supplemental methods.

## Results and discussion

**Fig. 1:**
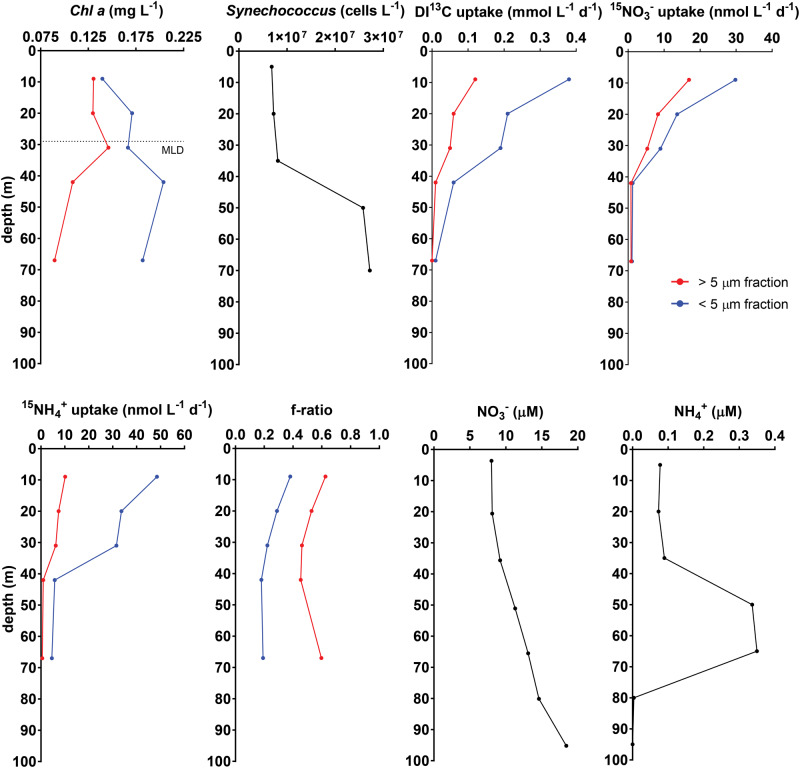
Depth distributions of biological, nutrient, and uptake rates at Station P, September 7th 2018. Chl a Chlorophyll a, ^13^C uptake radiolabeled carbon uptake rates, ^15^NO_3_^−^ uptake nitrate uptake rates, NH_4_^+^ uptake ammonium uptake rates. f-ratios calculated from the upper 100 m of the water column for the >5 µm (red) and <5 µm (blue) phytoplankton fraction. Nitrate (NO_3_^−^) and ammonium (NH_4_^+^) concentrations. Average mixed layer depth (MLD) for the cruise (29 m +/− 4.5 m) is indicated in *Chl-a* graph.

Station P is a low productivity system with high nitrate (7–15 µM) and low iron (<100 pM) concentrations characteristic of HNLC regions [13, 38, 39]. Phytoplankton blooms are rare, and primary production is sustained primarily by intrusion of nutrients from the shallow seasonal pycnocline [40]. Fitting with previous observations, phytoplankton at Station P during our sampling consisted primarily of small cells (<5 µm), including *Synechococcus*, small pennate diatoms, and autotrophic flagellates, with low abundance of large (>5 µm) flagellates and diatoms (Fig. 1). Correspondingly, the small size fraction had the highest chlorophyll concentrations, carbon, nitrate, and ammonium uptake rates, and represented 68% of total primary production (Fig. 1). The f-ratios (fraction of total primary production fueled by nitrate: here nitrate uptake/[nitrate + ammonium uptake]) were low for both size fractions, though the small cells had f-ratios half that of the large cells (Fig. 1). Together, the relatively low primary production and f-ratios observed at Station P indicate a system driven by regenerated production, particularly by small phytoplankton cells [24, 26, 41].

The *Synechococcus-*dominated deep chlorophyll maximum (DCM) was located at 50–70 m, below the mixed layer but above the ferricline, which was around 200 m (Supplementary Fig. S1). Additionally, iron inputs from dust deposition and mesoscale eddy events are infrequent at Station P compared to the adjacent subtropical North Pacific Gyre, and surface dissolved iron concentrations were low (<0.03 nM in 14 of 24 surface samples collected during the cruise; seasonally: ~0.05 nM spring and summer, ~0.1 nM winter) [41–45]. Ammonium and nitrate concentrations were relatively high at all sampled depths, suggesting neither oxidized nor reduced forms of nitrogen are limiting (Fig. 1 and Supplementary Fig. S2).

**Fig. 2:**
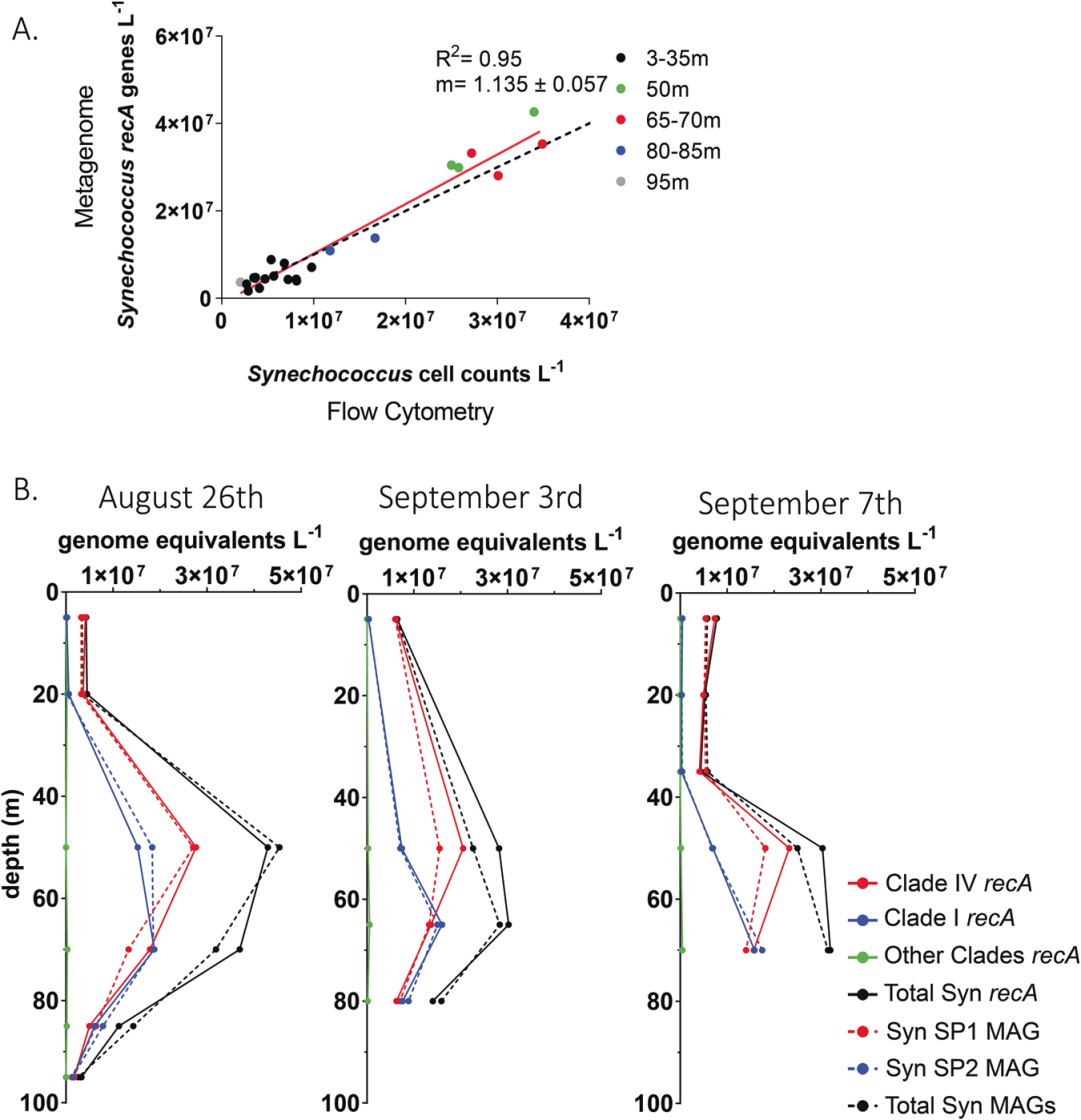
Metagenome-derived volumetric abundances of total *Synechococcus* cells and *Synechococcus* MAG populations at Station P. **A** Comparison of flow cytometry versus metagenome derived *Synechococcus* abundances. The red line is the linear regression model, and the black dashed line is the 1:1 line. **B** Metagenome-derived depth distributions of total *Synechococcus* cells, the subset of *Synechococcus* Clade I and IV populations, and the abundances of cells represented by the three different MAGs. *recA* abundances (solid line) and MAG abundances (dashed line) are separated into total (black), Clade I (blue), Clade IV (red), and Other Clades (green).

We used quantitative metagenomics to enumerate *Synechococcus* abundances at Station P. Genome equivalents were enumerated by identifying single copy recombinase A (*recA*) genes in a metagenome sample, and then converted to volumetric abundances via recovery ratios derived from internal standards added prior to extraction [36, 45–50]. A comparison between our quantitative metagenome-derived *Synechococcus* abundances and simultaneously collected flow cytometry *Synechococcus* cell concentrations show strong agreement (Fig. 2A), further supporting the use of internal standard quantitative metagenomics for determining absolute abundances of bacterial groups *in situ*. *Synechococcus* were the most abundant cyanobacteria at all depths (Fig. 2B), with peak densities of 5 × 10^7^ cells L^−1^ at 50–75 m (Fig. 2B). Taxonomic classification of the *recA* genes revealed the *Synechococcus* community was dominated by two populations, Clade I and IV, both previously found to be abundant at other high-latitude sites [18, 22, 51, 52]. These two clades represented >99% of the Station P *Synechococcus* population at all depths, with Clade IV most prevalent at 50 m and Clade I at 70 m.

Assembly of short and long reads produced two high quality *Synechococcus* genomes (Syn-SP1 and Syn-SP2) representing the two dominant clades (Table 1). Syn-SP1 is most closely related to a Single cell Amplified Genome (SAG; *Synechococcus C sp003208835; 96.5 ANI*) collected from 65 m in the subtropical North Pacific and isolates CC9902 and BL107 from *Synechococcus* Clade IV (87.6 and 87.1 ANI respectively) [53]. The second *Synechococcus* MAG (Syn-SP2) is phylogenetically distant from Syn-SP1 (77.6% ANI) and a member of Clade I that is closely related to SAG *Synechococcus*_C sp002500205 (90.9 ANI) and isolates CC9311 and WH8020 (87.9 and 87.6 ANI respectively). Syn-SP1’s genome is similar in size to other known Clade IV members (estimated 2.09 Mb at 100% complete, compared to 2.23–2.29 Mb for complete Clade IV genomes), while Syn-SP2’s genome is smaller than other Clade I members (estimated 2.26 Mb genome at 100% complete, compared to 2.53–2.70 Mb for complete Clade I genomes) [54]. These MAGs are the first genomes from *Synechococcus* clades known to dominate these high-latitude regions as identified with single gene analysis [19, 52].

**Table 1:** Genome characteristics of the *Synechococcus* Station P (Syn SP) MAGs.

MAG	Source samples	Clade	Assembled genome size (Mbp)	Completeness (%)	Contamination (%)	#Contigs	GC%
Syn_SP1	0–95 m	IV	1.99	95.29	0.27	116	54.87
Syn_SP2	0–95 m	I	2.21	97.92	1.64	81	53.4

Clade: *Synechococcus* clade MAG binned to within GTDB.

### Absolute quantification of MAG populations

We estimated the volumetric abundances (genomes L^−1^) of the *Synechococcus* MAG populations by deriving a coverage-based recovery ratio from the internal standard genome reads. Concentrations of the MAG populations were 3–6 × 10^6^ genomes L^−1^ at the surface, and 2–4 × 10^7^ genomes L^−1^ at the DCM (50–70 m) (Fig. 2B). Summed, the two MAGs accounted for nearly all *Synechococcus* genome abundances, as determined by either metagenome-derived *recA* counts (MAGs were 96% of total *Synechococcus recA)* or flow cytometr*y* (97% of *Synechococcus* flow cytometry counts). The Syn-SP1 MAG accounted for an average of 93% of the clade IV population. The Syn-SP2 MAG accounted for an average of 106% of the clade I population. We further validated the dominance of the SP1 and SP2 populations by mapping the metagenome reads to the MAGS and found 95% of unassembled *Synechococcus* metagenome reads mapped to the two MAGs. The MAGs thus represent the dominant *Synechococcus* populations and their genomic composition at Station P during our sampling.

### Adaptations of *Synechococcus* MAGs

**Fig. 3:**
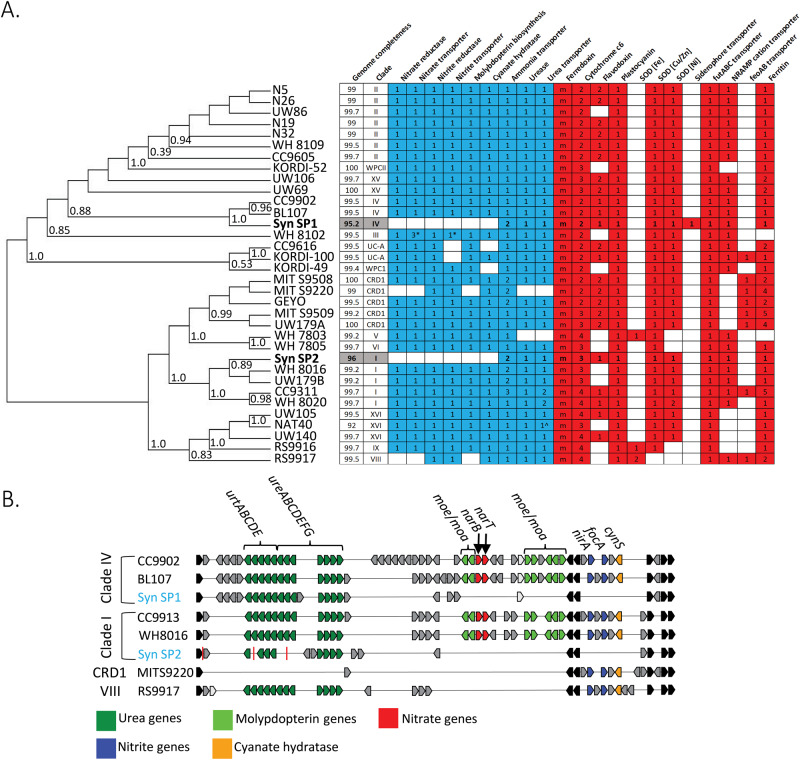
Comparison of marine *Synechococcus* nitrogen and iron utilization strategies. **A** Phylogenomic distribution of nitrogen acquisition (blue) and selected iron requiring genes (red) across *Synechococcus* clades. Phylogenetic relatedness was determined by multiple sequence alignment of each genome’s concatenated 120 bacterial marker genes in GTDB-TK. Station P *Synechococcus* MAGs are bolded and highlighted in gray. Numbers in a column represent gene copies in a genome, with m standing for multiple copies of the ferredoxin gene, and ^ standing for partial presence of the gene pathway. Only genomes with an estimated completeness of >90% are included here (for a complete set of genomes see Supplementary Fig. 3). **B** Distribution of nitrogen assimilation genes across select *Synechococcus* genomes. Station Papa MAGs are labeled in blue. Light gray indicates homologous genes that are present in different order within gene region. Red vertical lines indicate contig breaks.

The Station P *Synechococcus* genomes encode several strategies to cope with low iron availability; strategies that are well distributed across *Synechococcus clades* (Fig. 3A and Supplementary Fig. S3). For iron transport, both genomes possess NRAMP Fe/Mn and *idiABC* iron transporters. In addition, Syn-SP1 contains an operon for importing iron-chelated siderophores. These siderophore transport genes have previously been identified in members of Clades II, III, IV, CRD2, and UC-B, though these genomes were not complete enough to be confidently compared in our analysis of gene presence/absence (Supplementary Fig. S3) [16, 20]. It is unclear whether these populations synthesize their own siderophores or can obtain siderophores released by other community members [55]. Both Station P genomes possess a single copy of the ferritin gene for iron storage and Fur iron regulatory system. Equatorial HNLC-associated *Synechococcus* clade CRD1 possess multiple ferritin genes, potentially as an adaptation to low iron availability [20]. The Station P genomes encode a suite of alternative, low iron-containing proteins for core photosynthesis and electron transport chain functions, in addition to their high-iron dependent counterparts. This includes flavodoxin as a ferredoxin substitute and plastocyanin as a cytochrome c6 substitute, and the presence of only superoxide dismutases that use copper and zinc or nickel cofactors instead of iron [56–58]. Overall, the Station P genomes encode multiple strategies for obtaining and conserving iron, but these strategies are not unique to them, rather most of them are broadly distributed in *Synechococcus* genomes obtained from both low and high iron environments.

The Station P *Synechococcus* genomes were unusual in their nitrogen assimilation pathway in that they are the first known Synechococcus genomes to be missing the entire nitrate/nitrite reduction pathway. Within a nitrogen gene cluster highly conserved among cyanobacteria, both genomes are missing genes for nitrate reductase, nitrite reductase, nitrate and nitrite transporters, cyanate hydratase, and the nitrate reductase cofactor molybdopterin biosynthesis genes (Fig. 3B). By contrast, the genomes both contain two distinct ammonium transporters, all urease subunits, and a urea ABC transporter. For the two ammonium transporters found in both Syn-SP1 and Syn-SP2, one is closely related to other *Synechococcus* ammonium transporters, and the other is closely related to euryarchaeal and Thermotogae ammonium transporters (Supplementary Fig S4). Both of these ammonium transporter types are present in closely related Synechococcus genomes that contain two ammonium transporters.

Support for the absence of nitrate and nitrite utilization genes from Station P *Synechococcus* populations is provided by (1) the high quality and completeness of the MAGs from multiple independent assemblies, (2) the missing nitrogen genes’ location in the interior of a contig flanked by nitrogen genes with homology to taxonomically related genomes, and (3) individual long Nanopore reads lacking these genes (Supplementary Fig. S5).

**Fig. 4:**
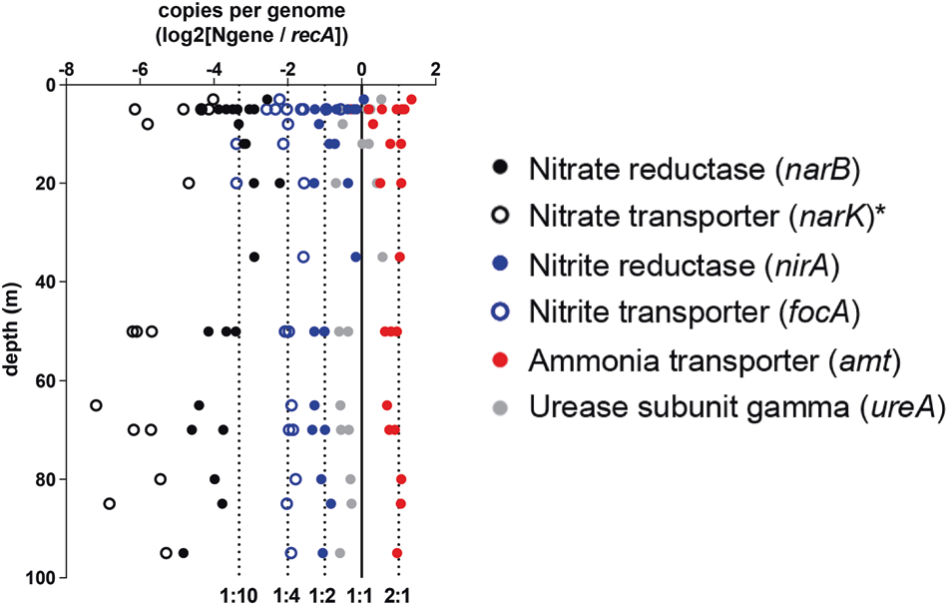
Depth distribution of nitrogen assimilation gene prevalence in Station P unassembled metagenomes. Nitrogen gene copy numbers per genome were calculated as the ratio of *Synechococcus* nitrogen gene read abundance to *Synechococcus recA* read abundance, and then log2 normalized. The solid vertical line is the 1:1 ratio representing a single copy of the gene per *recA* (*Synechococcus* genome equivalent), with dotted lines representing other genome copy numbers. *Seven samples are not included due to no nitrate transporter reads found in those samples.

To further increase confidence that nitrate and nitrite assimilation genes are absent in Station P *Synechococcus* populations, we determined *Synechococcus* nitrogen gene copy numbers in the unassembled metagenomes. If the majority of Station P *Synechococcus* genomes possess nitrate reductase (*narB*), then *narB* copies per genome should be approximately one (Fig. 4). Instead, we found *Synechococcus narB* copy numbers much less than one (range 0.04–0.26), supporting their depletion in Station P *Synechococcus* populations, with the nitrate reductase genes present mostly belonging to Clade IV. Nitrite reductase (*nirA*) gene ratios were higher (range 0.42–1.35), though this may be due to misannotation given *nirA*’s high sequence similarity to sulfite reductase. Copy numbers for the nitrate transporter *narT* and the nitrite/formate transporter *focA* were also much lower than one, supporting depletion in the Station P *Synechococcus* populations. *focA* gene abundance was approximately 25% for the *Synechococcus* community, with nitrite transporters from both Clades I and IV, so it is likely that at least two *Synechococcus* variants with nitrite reductase represent a fraction of the station P population. Based on this data, it appears that, though the Synechococcus backbone represented by our Syn-SP1 and SP2 genomes represent ~97% of the Synechococcus population, at least a few other ecotypes sharing this backbone are present within this community that contain a whole or partial nitrate reduction pathway and represent up to a quarter of the total Synechococcus population at Station P. These other ecotypes may be present at higher abundances during other seasons, such as during the small spring phytoplankton bloom that occurs at Station P. By contrast, *Synechococcus* ammonium transporter (*amt*) genome copy numbers were between two and three across all Station P samples (range 0.91–2.77), consistent with the two *amt* copies in our *Synechococcus* genomes.

The Station P genomes are the first known *Synechococcus* to lack both nitrate and nitrite uptake and utilization genes. Two previously sequenced *Synechococcus* genomes do not encode the ability to use nitrate but are capable of nitrite assimilation: MIT S9220 isolated from an HNLC zone in the Equatorial Pacific, and RS9917 isolated from the Red Sea [59–61]. Both isolates were obtained using media with ammonium as the nitrogen source. The loss of both nitrate and nitrite assimilation therefore may be specific to high latitude HNLC *Synechococcus*, though more metagenome sequencing and cultivation on non-traditional *Synechococcus* media (with ammonium used as N source instead of traditionally used nitrate) is needed [62, 63].

The absence of nitrate and nitrite assimilation and the enrichment of ammonium transporters suggests the dominant *Synechococcus* populations at Station P cannot utilize nitrate or nitrite as a nitrogen source and instead rely on ammonium or other reduced forms of recycled nitrogen for growth. Despite high concentrations of nitrate in this region, the benefit to losing nitrate and nitrite assimilation is a decreased cellular iron and energy demand. Reduction of exogenous nitrate and nitrite to ammonium requires a large quantity of iron, with nitrate reductase containing four iron atoms per enzyme and nitrite reductase containing five iron atoms [11, 64]. In many areas of the ocean, iron stress is coupled with nitrogen stress leading cyanobacteria to maintain assimilation capabilities for all sources of N, including nitrate and nitrite, and their associated high iron cost [65]. However, in HNLC regions such as Station P, abundant N sources, particularly ammonium and urea, may drive the system more heavily towards iron limitation, resulting in evolutionary pressure to prioritize iron conservation over nitrate utilization via loss of the nitrate/nitrite assimilation pathway [66].

### Global patterns in nitrate and nitrite assimilation loss

**Fig. 5:**
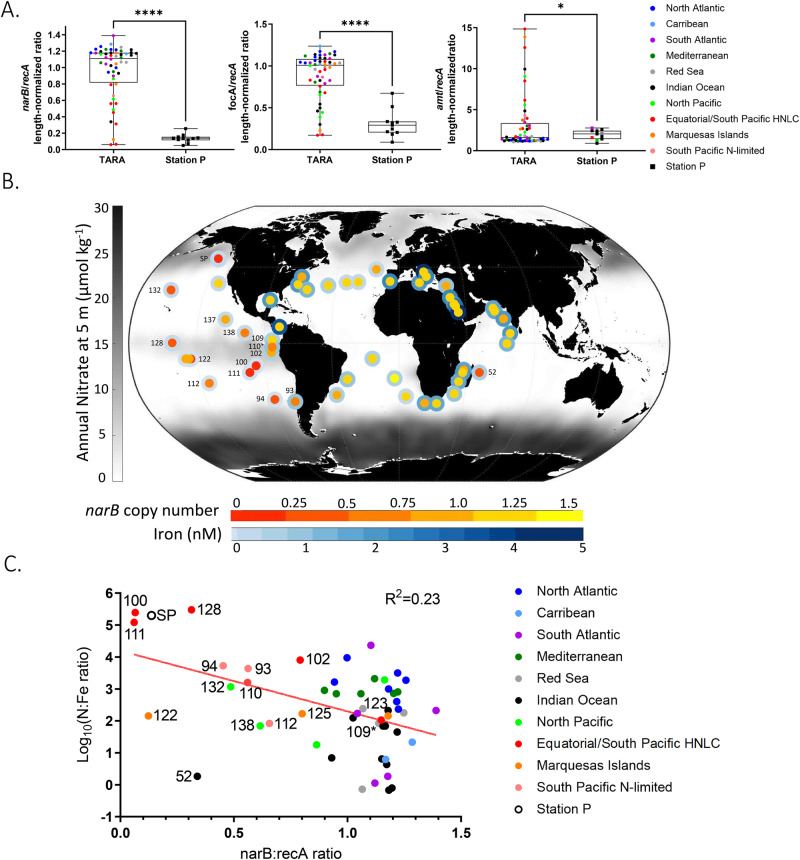
Global prevalence of *Synechococcus* select nitrogen metabolism genes in relation to iron and nitrogen standing stocks. **A** *Synechococcus g*ene copy numbers for nitrate reductase, nitrite/formate transporter, and ammonium transporters for all TARA Stations and all EXPORTS Station P samples at 5 m depth. Significance of *p*-value of Welch’s t test signified by *(*p* < 0.05) and ****(*p* < 0.0001). **B** Global patterns of nitrate (background map), dissolved iron concentrations (outer circle of each datapoint), and *Synechococcus* nitrate reductase genome copy numbers (inner circle of each datapoint) for TARA stations and Station P (SP). *Synechococcus* nitrogen copy numbers were calculated using gene length normalized ratios derived from the TARA dataset. Annually averaged nitrate concentrations (µmol kg^−1^) were derived from the WOA2018 global dataset. Dissolved iron concentrations (nM) determined from the PISCES biogeochemical model and BYONIC 3 R biogeochemistry dataset. **C** Log_10_ normalized nitrate to iron ratio versus *Synechococcus* nitrate reductase gene copy number of the TARA stations and EXPORTS Station P. Red line indicates the linear regression (R^2^ = 0.23, *p*-value = 0.0004). Select stations within the Pacific and Indian Oceans are labeled with their TARA ID.

To examine whether *Synechococcus* nitrate and nitrite assimilation loss is widespread in the global ocean, we extended the nitrogen gene copy number analysis to the TARA Oceans metagenomes. TARA Oceans metagenomes from the 0.2–3.0 µm size fraction were annotated via BLASTX searches to identify *Synechococcus* nitrate reductase (*narB*), nitrite transporter (*focA*), ammonium transporter (*amt*), and recombinase A (*recA*) gene hits in order to calculate nitrogen gene copy numbers per genome equivalents across the global ocean. The ratios at each station were then compared to surface nitrate and dissolved iron concentrations (N:Fe ratios, or nanomolar nitrate divided by nanomolar iron) predicted by the PISCES global ocean biogeochemical model [67, 68]. Station P *Synechococcus narB* and *focA* genome copies were depleted compared to most TARA stations (Fig. 5A). By contrast, *Synechococcus* ammonium transporters (*amt*) were typically found at greater than one copy per genome at both Station P and TARA stations, though there was a statistically significant enrichment at Station P (Fig. 5A).

Based on the TARA metagenome and PISCES nutrient data, nitrate reductase (*narB)* copy numbers are low in the HNLC regions of the southeastern and equatorial Pacific that have high nitrate and low chlorophyll *a* concentrations (Fig. 5B, C; linear regression, *p* < 0.05, *R*^2^ = 0.23). Similar to Station P, HNLC zones also have low nitrite transporter (*focA*) and high ammonium transporter (*amt*) copy numbers (Supplementary Fig. S6A). Additionally, principal component analysis (PCA) was used with the three nitrogen gene copy numbers and the N:Fe ratios as variables. The PCA analysis showed that Station P clusters with Station 128, the most isolated Equatorial Pacific HNLC Tara Station, while other HNLC stations clustered away from the majority of other TARA Stations Supplementary Fig. S6B). Two outliers, Station 52 (Southwest Indian Ocean) and Station 122 (East of Marquesas Islands), also had low *narB* copy numbers despite having low predicted annual N:Fe ratios. However, modeled nutrient profiles of these two regions suggest they are iron-limited for large parts of the year, with iron inputs occurring seasonally, and thus the annual modeled N:FE ratio may be too broad to capture the complexity of nutrient limitation at these sites [69, 70]. Aside from these outliers, the lowest *narB* copy numbers correspond with Station P and the TARA Stations with the highest N:Fe ratios, all of which are within or adjacent to HNLC regions, supporting nitrate assimilation gene loss is linked to iron limitation (Fig. 5C).

## Synthesis

Our findings suggest the dominant *Synechococcus* at Station P are likely incapable of assimilating either nitrate or nitrite and instead rely on reduced nitrogen sources such as ammonium and urea. Traditionally, nitrate and nitrite assimilation has been considered to be a core trait of *Synechococcus*, as they are known to possess the entire nitrate assimilation pathway and exhibit cellular heterogeneity of nitrate uptake as an adaptation to fluctuating nitrate availability [71]. However, in iron-limited HNLC zones, these genes may be less advantageous, with the loss of genes for nitrate and/or nitrite assimilation eliminating the cellular iron demand required by their respective reductases. The f-ratios were low at Station P, particularly in small cells below the mixed layer where *Synechococcus* is most abundant, which could be attributed in part to the lack of *Synechococcus* nitrate and nitrite uptake capabilities.

The TARA Oceans nitrogen gene analysis indicates *Synechococcus* nitrate reductases are also depleted within the equatorial and sub-tropical Southern Pacific HNLC regions as well as in a portion of the Southwestern Indian Ocean, with nitrite transporters also being as depleted as Station P in the most isolated portion of the equatorial Pacific HNLC zone (TARA Station 128). Conversely, ammonium transporters are enriched in Pacific HNLC zones and at Station P. The results suggest having both nitrate and nitrite assimilation capabilities, or nitrite assimilation alone, is not a core trait of marine *Synechococcus*. Isolation of these HNLC Synechococcus and in vivo investigations of their iron quotas and growth on various nitrogen compounds is necessary to confirm this hypothesis [59–61].

These new *Synechococcus* genomes display signs of genomic streamlining and iron-scavenging strategies that may aid their survival in the iron-limited environment at Station P. *Synechococcus sp*. SP1 has an estimated 6% smaller genome compared to close Clade IV relatives, while SP2 has an estimated 13% reduction in genome size compared to its Clade I relatives. The smaller genomes reduce the overall nitrogen and phosphorus requirements for genome maintenance and replication, which could be an important means of reducing macronutrient quotas when iron limitation prevents their uptake and utilization. *Synechococcus* *sp.* SP1 also possesses a putative siderophore uptake system, while SP2 does not appear to possess one. Interestingly, unlike recently identified siderophore-possessing *Prochlorococcus*, which tend to be low light and low temperature adapted strains (HLI and LLI ecotypes) that reside primarily at the deepest chlorophyll maximums, SP1 at Station P has siderophore uptake and resides at the surface as well as down to 70 m. Meanwhile, the more deepwater associated SP2 lives at 50–85 m with no detected siderophore uptake system [16]. This difference in vertical organization of siderophore uptake gene presence might be due to the difference in the dissolved iron depth profile, with iron concentrations being depleted at the surface and increasing with depth where SP2 is most abundant, forgoing the need for siderophore uptake by this strain.

The disparate loss of nitrogen assimilation genes across the *Synechococcus* phylogeny suggests multiple factors might be influencing their retention. If iron limitation exerts a significant selective pressure for nitrogen gene loss, whether that be directly as an iron-limitation strategy or indirectly due to iron-limitation causing the buildup of reduced nitrogen that forgoes the need for nitrate assimilation, why have high latitude HNLC *Synechococcus* lost both nitrate and nitrite assimilation while equatorial HNLC *Synechococcus* strains only abandon a portion of the pathway? This may be due to dueling pressures between iron stress and competition for reduced nitrogen species. Complete loss of nitrate and nitrite assimilation may only be possible at high latitudes where there is reduced competition for ammonium from other cyanobacteria. *Prochlorococcus* is a major competitor for reduced nitrogen species, often relying solely on ammonium as their nitrogen source. They are dominant in many low to mid latitude regions but absent at high latitudes [3, 6, 11, 72], including Station P where they are undetectable by pigment analysis, flow cytometry, and metagenomics. In low latitude HNLC regions, competition with *Prochlorococcus* and heterotrophic bacteria may result in *Synechococcus* at least maintaining nitrite uptake. In high latitude HNLC regions, competition for ammonium is lower due to the absence of *Prochlorococcus*. Further, overall community competition for ammonium is likely low at Station P given the relatively high standing stocks of ammonium at the DCM; Station P’s peak ammonium concentration was greater than threefold higher than those measured in equatorial HNLC sites (Supplementary Fig. S7).

The model for *Prochlorococcus* nitrogen gene gain and loss appears to be a close counterpart to *Synechococcus*, where nitrogen gene retention is selected for by nitrogen competition but selected against in iron- or light-limiting environments [11, 73]. Low-light *Prochlorococcus* typically possess only nitrite assimilation genes and are abundant at the subsurface nitrite maximum at the base of the euphotic zone [72]. Competition for ammonium near the nitrite maximum is intense due to the presence of ammonia-oxidizing archaea, driving low-light *Prochlorococcus* to acquire nitrite generated by ammonia oxidizers [74–76]. Additionally, most low-light *Prochlorococcus* are restricted to the base of the euphotic zone, where the energy required to reduce nitrate may drive selection against maintaining nitrate assimilation [11, 72]. High-light *Prochlorococcus* suffer less light limitation and many strains possess both nitrate and nitrite assimilation pathways [6, 11, 77]. Critically, high-light *Prochlorococcus* clades adapted to low-iron conditions in the Equatorial Pacific HNLC zone do not contain nitrate and nitrite utilization genes, reducing their iron requirements [11, 18, 78].

The reduced ability to assimilate nitrate and nitrite by some *Synechococcus* populations suggests a re-evaluation of their role in HNLC nutrient cycles and carbon export. In these regions, iron availability limits primary production and nitrate utilization due to the iron requirements for their respective enzymes. New iron delivered to this region via lateral advection, entrainment from subsurface waters, or from atmospheric inputs leads to pulses of nitrate-based new production typically dominated by large phytoplankton (i.e., diatoms) that exceed surface remineralization and can be exported [79]. However, according to our findings, any new inputs of iron would not stimulate nitrate utilization and subsequent nitrate-fueled growth in the dominant *Synechococcus* population at Station P, prohibiting them from significantly contributing to organic carbon export fluxes. Instead, these Station P *Synechococcus* strains are confined to a tight recycling loop where they are dependent on other primary producers to assimilate nitrate, which then directly or indirectly regulates the subsequent availability of recycled forms of nitrogen such as ammonium or urea. The direct inability of these *Synechococcus* strains to contribute to new production means their fixed carbon is continuously cycled in the surface ocean when grazed upon or remineralized by bacterioplankton, leaving little carbon to be exported to depth. Thus, whereas small cyanobacteria such as *Prochlorococcus* and *Synechococcus* can be major contributors to ocean carbon export, in the North Pacific and possibly other HNLC regions, this may not always be the case [1, 6, 80, 81].

It should be noted that these strains may not dominate the *Synechococcus* community at Station P throughout the year. While our analysis suggests the dominant *Synechococcus* populations at Station P do not contain nitrate and nitrite assimilation pathways, we did detect the presence of these genes at relatively low levels (<10% of genome equivalents for *narB*, and ~29% of genome equivalents for *focA*) in the unassembled metagenome. This suggests there is a small population of nitrate/nitrite and nitrite utilizing *Synechococcus* ecotypes maintained in the community. At different times of the year these populations may increase in abundance, potentially when iron is pulsed into the system, altering nitrogen cycles and carbon export potential by the *Synechococcus* community at Station P. Alternatively, these nitrate/nitrite utilizing ecotypes could persist at lower abundance year round and compensate for the increased iron quota of these genes through some other iron conservation strategies, such as iron storage, iron uptake, or transcriptional controls on their nitrogen assimilation machinery. This emphasizes the need for further genomic and biogeochemical investigations to model the role of cyanobacteria more accurately in the Fe, N, and C cycles of HNLC zones. Further, if nitrogen assimilation adaptation driven by iron limitation is prevalent within other HNLC *Synechococcus* populations, as our TARA Oceans analysis suggests, it has significant impacts on nutrient cycling and associated carbon export by the cyanobacterial community in a sizable fraction of the global ocean. Overall, our results further support how iron availability affects primary productivity directly through limitation but also has fundamentally shaped phytoplankton functional capabilities leading to cascading effects on marine biogeochemical cycles and food webs.

### Supplementary information

Supplemental Material

## Data Availability

All metagenomes have been submitted to NCBI under BioProject PRJNA785333 (Biosamples SAMN24695624-SAMN24695646.)
