# Supplementary material for: Synechococcus nitrogen gene loss in iron-limited ocean regions
Source: ISME Commun. 2023 Oct 2;3:107. doi: 10.1038/s43705-023-00314-9 (PMC10545762; doi:10.1038/s43705-023-00314-9)
Supplement: Supplementary file 1 — Supplemental Material [file 43705_2023_314_MOESM1_ESM.docx]

**SUPPLEMENTAL MATERIALS and METHODS**

**Sample Collection and Preservation.** Samples were collected at Station P in the North Pacific from August 18th to September 7^th^, 2018 as part of the NASA Export Processes in the Ocean from RemoTe Sensing (EXPORTS) expedition (1). Discrete water samples from 5 to 95 m were collected using a CTD/rosette and dispensed into acid-washed 4 L bottles. For each sample, 1 to 2.5 L of water was peristaltically pumped through a 25 mm diameter 5.0 µm pore TMTP Isopore filter and a 25mm diameter 0.2 µm pore PES. Filters were placed into a 2 mL cryovial using sterilized forceps and flash-frozen in liquid nitrogen. Profile sampling for dissolved iron (Fe) was conducted with a trace metal clean rosette system. Dissolved Fe samples were filtered (<0.2 µm, Pall Acropak), collected in trace metal clean low density polyethylene bottles, acidified to pH <1.8 with ultrapure hydrochloric acid (Fisher Optima), and stored at room temperature until analyzed at the University of South Florida (2).

**Dissolved Iron (Fe) Concentration Measurements.** The concentration of dissolved Fe in project samples was measured by inductively coupled plasma mass spectrometry (ICP-MS, ThermoScientific Element XR) at the University of South Florida following preconcentration with a seaFAST-pico (Elemental Scientific) system (3). Iron concentration data for Station Papa is available in the Biological and Chemical Oceanography Data Management Office (BCO-DMO) database (Project#757837 Dataset#869683) (4).

**Flow Cytometric Determinations of *Synechococcus*.** Analysis of seawater samples for phytoplankton communities were performed on a Becton Dickinson Influx Cell Sorter (BD-ICS) flow cytometer while at sea following previously published protocols (5). Briefly, the ICS used a blue laser (488 nm) and two detectors for fluorescence (692 ± 40 nm and 530 ± 40 nm) and two for scattering properties (forward scatter and side scatter). The ICS optical alignment was performed daily with fluorescent beads (Spherotech, SPHEROTM ~3.0 μm Ultra Rainbow Calibration Particles) following standard protocols and photomultiplier tube (PMT) gains were identical for all samples to minimize artifacts due to instrument settings. Samples for flow cytometric analysis were collected from CTD rosette mounted Niskin bottles and analyzed within ~30 min of collection. At Station P, an average of >5000 phytoplankton cells were interrogated per sample and specific groups were identified based on light scattering and fluorescence properties. *Synechococcus* have a distinct scattering and fluorescence signature that allow these autotrophic cyanobacteria to easily be identified in relation to other microalgal groups. Cell concentrations are calculated from cell counts determined over a known amount of time (cells per second) and a sample flow rate (volume per second) determined for each set of samples collected from each CTD cast.

**Isotopic Uptake Incubations.** Triplicate samples for carbon and nitrogen stable isotope incubations were collected via a trace metal clean CTD rosette in 1 liter acid-cleaned polycarbonate bottles. Each bottle was spiked with a dual inoculation of NaH^13^CO_3_ isotope and Na^15^NO_3_^-^ isotope at a concentration of approximately 10% of ambient concentrations of DIC and NO_3_^-^, respectively. Bottles were incubated for 24 hours. Following incubation, two of the triplicate samples were gravity filtered through a 5 µm polycarbonate filter with the filtrate subsequently being vacuum filtered through a pre-combusted Whatman glass fiber filter (GF/F) to collect size fractionated samples. Sample material retained on the polycarbonate filter was washed onto a new, pre-combusted GF/F using filtered (0.2 µm) seawater. One sample was retained as a whole sample and filtered through a pre-combusted GF/F. Filters were dried and stored until onshore preparation and analysis at the UC Davis Stable Isotope Facility. Ambient nutrient concentrations used in this analysis were collected according to Siegel et al. (2021). For further details on carbon and nitrogen stable isotopic uptake methods refer to Meyer et al., *in revision*.

**DNA Extraction and Internal Standard Addition.** DNA was extracted from the filters using a DNeasy Powerwater kit (Qiagen, Hilden, Germany). The 0.2 µm filters were removed from the storage tubes using sterilized forceps and placed in Powerwater bead tube. PW1 lysis buffer was added to the original filter storage tube, vortexed briefly, and then transferred to the Powerwater bead tube to ensure complete transfer of sample. For quantitative analysis, three bacterial genomic internal standards (*Blautia producta*, *Deinococcus radiodurans*, and *Thermus thermophilus*), were individually added at 4 ng each (previously quantified by PicoGreen) in 25 µl volumes to the Powerwater bead tube prior to starting the DNA extraction, aiming for 1% of total DNA being comprised of genomic standards and assuming 1000 ng native DNA in the sample. The bead tube was shaken with a vortex adapter for 5 minutes. The remainder of the extraction followed the kit manual, except for an additional ethanol removal step where the filter column was transferred to a clean 2mL microcentrifuge tube and spun at 13,000 rcf for 2 minutes to remove trace ethanol prior to eluting the DNA. The DNA was eluted in 100 µl of elution buffer (10 mM Tris, pH 8.0). DNA yields were quantified with the Quant-iT PicoGreen dsDNA kit (Molecular Probes Inc., Organ, United States).

**Library Preparation and Sequencing.** Metagenomic libraries were prepared using the KAPA HyperPlus Library Preparation Kit (Kapa Biosystems Scientific, Massachusetts, United States). 100 ng of purified DNA diluted in 10 mM Tris-HCL was used as input. For the fragmentation step, a 23 minute fragmentation time at 37 °C produced an average DNA fragment size of 330 bp (including ligated adapters). Samples were barcoded via the addition of a NimbleGen SeqCap Adapters (Roche NimbleGen Inc., Wisconsin, United States; containing single index barcodes) at a concentration of 1 µM. Reaction cleanup steps were conducted using AMPure XP reagent beads (Beckman Coulter, California, United States) with a 0.8X SPRI bead cleanup following adapter ligation and a 1X cleanup following library amplification. Additionally, a dual-SPRI bead size selection selecting for 400 bp fragments was performed after the post-ligation cleanup. Five cycles were used for the library amplification reaction. Library fragment composition and quality was analyzed on the Agilent 2100 Bioanalyzer with High Sensitivity DNA chip (Agilent Technologies, Waldbronn, Germany) and quantified with the Quant-iT PicoGreen dsDNA kit (Molecular Probes Inc., Organ, United States). Equimolar concentrations of the barcoded libraries were then pooled and sequenced using HiSeq 4000 platform 150 bp PE (Illumina, San Diego, CA).

**DNA extraction for long read sequencing.** A phenol chloroform extraction was performed on one sample (65 m, September 5^th^) to generate long DNA fragments for Oxford Nanopore sequencing. Cells on the filter were resuspended in 500 µl of T_50_E_50_ (50 mM Tris pH 7.5, 50 mM EDTA pH 8) and frozen at -80°C overnight with the filter remaining in the tube. Lysis was performed via the addition of 25 µL lysozyme (10 mg lysozyme per 1mL 10mM Tris, pH 7.5) to the tube with the frozen pellet and thawed in a room temperature water bath. The cell lysate was frozen again at -80°C overnight and thawed the following day in a room temperature water bath. Protein was degraded by adding 100 µl STE (0.5% SDS in T_50_E_50_) and 35 µl of proteinase K (2 mg proteinase K per 1 mL DNase-free water) and incubating for an hour at 55˚C with occasional mixing. RNA was removed via the addition of 5 µl RNase A (10 mg RNase A per 1 mL DNase-free water) and incubating for 30 min at 37°C, with mixing by inversion done at 10-minute intervals. Deproteinization was carried out through the addition of 200 µl of 5 M sodium perchlorate. 640 µl of Tris-equilibrated phenol:chloroform (1:1 mixture of phenol and chloroform with phenol buffered to pH 7.8-8.0) was added to the sample, mixed by inversion and separated into the organic and aqueous phases via centrifugation at 12,000 rpm for 10 minutes. The aqueous phase was transferred to a new tube, and the phenol:chloroform separation was repeated. The aqueous phase was once again transferred to a new tube, and 600 µl chloroform was mixed into the aqueous phase, followed by centrifugation at 12,000 rpm for 10 minutes. The aqueous phase was transferred to a new tube, and the chloroform addition and centrifugation was repeated. To concentrate the extracted DNA, the aqueous phase was transferred to a new tube, and a 1/10^th^ volume of 3 M sodium acetate was mixed in with the aqueous layer. Two volumes of cold 100% ethanol were mixed with the sample, and the reaction placed on ice for 30 minutes. The precipitated DNA was pelleted by centrifugation at 12,000 rpm for 10 minutes. The supernatant was removed by pipette. One milliliter of 70% ethanol was added to the pellet, spun briefly, and removed by pipette. The pellet was air-dried and then resuspended in 50 µL of molecular-grade water. DNA concentration was quantified as 5.5 ng µL^-1^ with the Quant-iT PicoGreen dsDNA kit (Molecular Probes Inc., Organ, United States), and the presence of larger fragments (~10kb) was determined with the Agilent High Sensitivity DNA kit (Agilent Technologies, Waldbronn, Germany). Forty microliters of this DNA sample was sent to the UNC High-throughput Sequencing Facility for sequencing on an Oxford Nanopore flow cell.

**Metagenome Read Processing**. For Illumina sequences, FastQC was performed to determine the general quality of the raw reads (6). Trimmomatic (version 0.39, paired-end mode) was used to remove adaptor sequences and low-quality base pairs (bp) from both the forward and reverse reads with a sliding window looked of 10 base pairs and trimming base pairs that had an average PHRED score of less than 20 (7). Trimmed reads < 50 bp were removed. Another FastQC was performed to determine the quality of the trimmed reads and ensure that adaptor sequences were successfully removed. Paired forward and reverse trimmed reads were merged using Pear (version 0.9.6, --p-value 0.01 --min-overlap 10 --min-asm-length 50 --min-trim-length 50 --quality-threshold 0 --max-uncalled-base 1.0 --test-method 1 --empirical-freqs --score-method 2 --cap 40) a minimum overlap of 10 bp to be successfully merged into one assembled read (8). The merged reads were quality checked with FastQC. The merged reads, unmerged forward reads, and unpaired forward and reverse reads that lost their paired read during trimming were all concatenated into one file to be used for read annotations. The concatenated file metagenome reads were then annotated with a DIAMOND search against the NCBI Refseq protein database (version 95; default settings) (9).

As described above, three genomic internal standards were added at a known concentration to each sample prior to DNA extraction. Each internal standard represents a genome alien to surface ocean communities. Internal standard reads were identified via a BLASTn search (megablast; e-value <0.001, >90% identity) of the processed metagenome reads against the internal standard genomes, and results filtered to retain hits that had a >95% identity to internal standard sequences. To identify the number of internal standard gene hits, a BLASTx of the BLASTn-identified reads was used. All confirmed internal standard reads were removed from the dataset before proceeding with analysis.

For the Nanopore reads, *NanoPlot* (version 1.38.0) was used to determine the quantity, size, and quality of reads (10). Sequencing adapters were removed from the reads with *PoreChop* (version 3). *Nanofilt* (version 2.0.0) was used to trim the edges of each read to ensure removal of adaptors (--headcrop 50, --tailcrop 50), and removed reads with an average quality score <8 and/or a length of < 100 bp (-l 100, -q 8) (7). Quality, quantity, and read length of the quality-controlled and Porechopped reads were assessed using *NanoPlot* (10).

**MAG assembly and annotation.** metaSPAdes (version 3.13.0) was used to assemble contigs from surface reads (all samples from 5-35 m) and deep reads (50-95 m) separately (11). Metagenome reads were mapped onto contigs with Bowtie2 (version 2.3.4) (12), and the resulting alignments were indexed and sorted using SAMtools (version 1.9) (13). Metagenome binning was then performed using MetaBAT (version 2.13), MaxBin (version 2.2.6), and CONCOCT (version 1.0.0) (14-16). Bins from the three binning programs were consolidated using DAS Tool (version 1.1.1), and the quality and general characteristics of each bin were assessed with CheckM (version 1.0.13) (17, 18). Taxonomic assignments were given to the MAGs (or bins) based on Average Nucleotide Identity (ANI) and placement on the reference phylogenomic tree using GTDB-Tk (version 0.3.2; Genome Taxonomy Database release 04-RS89) (19).

To improve the quality of the *Synechococcus* MAGs, several additional assemblies were performed: 1) nanopore deep and surface samples reads, 2) *Synechococcus*-annotated Illumina reads with *Synechococcus*-annotated nanopore reads, and 3) Illumina reads from individual samples. For MAG Syn_SP1, we used minimus2 from the AMOS toolkit (version 3.1.0) to further assemble the bin reconstructed from the *Synechococcus*-annotated Illumina with nanopore assembly (min identity 0.94; min overlap 40bp) (20). For Syn_SP2, we ran minimus2 on the bins from the *Synechococcus* Illumina with nanopore assembly and the deep assembly. We specifically focused on the nitrogen assimilation gene cluster region. However, SP2’s N gene region was still broken into 3 contigs despite the fact that nanopore long reads spanned across them and suggested there were 2 ~400bp gaps. We then manually pulled contigs from the deep samples with nanopore assembly and the FN446 assembly (single sample) to fill in those gaps. Contigs that did not get assembled by minimus2 were mapped onto the assembled contigs with minimap2 and visualized with IGV (21, 22) to identify and remove redundant contigs. The resulting bins were visualized with Anvi’o (v5) to inspect contigs’ coverage and composition (23). After manual curation, the MAGs were assessed by checkM to see if quality had improved.

For each bin, protein-coding genes were identified and annotated with Prokka (version 1.12, gene calls made using Prodigal version 2.6) (24). Blastp searches were performed against the Prokka protein sequence files using query sequences related to nitrogen and iron metabolism to identify the functional capabilities of the *Synechococcus* MAGs.

***recA* quantification and taxonomy**. *Synechococcus* genome equivalents for each sample were estimated with absolute *recA* gene count. The *recA* gene encodes a DNA repair protein recombinase A and is a well-described single-copy gene. To identify *recA* genes in the metagenome samples, a protein database containing viral sequences and sequences annotated with the key words “recombinase RecA,” “protein RecA,” “recombinase A,” or “RecA protein” was assembled from the NCBI RefSeq protein database (v.95). Metagenome reads were then compared to this custom database using a DIAMOND homology search. To reduce the chance of getting false positives, top hits with a bit score >50 were counted as a recA gene only if the read was also annotated as a *recA* gene in the homology search against the NCBI RefSeq database. To directly compare *recA*-based abundances with MAG abundances, the recA reads were re-annotated with a GTDB RecA database containing RecA protein sequences from the GTDB representative genomes and the *Synechococcus* MAG RecA sequences (25).

The resulting *recA* read counts for each *Synechococcus* clade were converted to volumetric *recA* abundances using equations described in (26, 27):

$$1) S_{r}=\frac{S_{S}}{S_{P}}$$

$${2) R}_{r}=\frac{S_{r}}{S_{a}}$$

$${3) G}_{a}=\frac{G_{S}}{R_{r}}$$

(1) S_r_: Copies of internal standard genome recovered in sequence library.

S_S_: protein encoding internal standard reads in the sequence library.

S_p_: protein encoding genes in the internal standard reference genome.

(2) R_r_: read-based recovery ratio. The proportion of standard molecules added that were sequenced.

S_a_: molecules of internal standard genome added to the sample.

(3) G_a_: Molecules in the sample of any gene category.

G_s_: total reads of any gene category in the sample sequence library

For each internal standard genome, the number of gene hits was divided by the total number of genes in the genome to get a value for the number of internal standard genomes recovered. To convert metagenome reads to absolute values, a conversion factor for a given sample was created by dividing the number of internal standard genomes added by the number of internal standard genomes recovered. The average conversion factor for a sample was derived from the average of the resulting three conversion factor values. *recA* reads recovered in the sample was then multiplied by this conversion factor and normalized by dividing by the volume filtered to get the value for absolute number of *recA* reads per liter, which is a proxy for cell abundance.

**Whole genome quantification.** *Synechococcus* MAG volumetric genome abundances were estimated with a coverage-based recovery ratio derived from the internal standard genomes with the following calculations:

$${4) R}_{cov}=\frac{S_{cov}}{S_{a}}$$

$$5) M_{a}=\frac{M_{cov}}{R_{cov}}$$

(4) R_cov_: coverage-based recovery ratio.

S_cov_: mean depth of coverage of internal standard genomes by metagenomic reads.

S_a_: molecules of internal standard genomones added to the sample

(5) M_a_: molecules of any genome (MAG) in the sample.

M_cov_: mean depth of coverage of any genome (MAG) in the sequence library.

Metagenomic reads were mapped onto the internal standard genomes with bowtie2 and the mean depth of coverage was calculated by dividing the total number of bases mapped by the size of the genomes (SAMtools bedcov) (12, 13). The mean depth of coverage represents the number of internal standard genome recovered through sequencing, and this was divided by the number of genomes added to the samples to get at the recovery ratio. The number of MAGs recovered from the sequence library was retrieved by mapping reads onto the MAG and calculating mean depth of coverage. The volumetric abundance of the MAGs was determined by normalizing MAG abundance in the sample (Ma) by the volume of seawater filtered.

**Genome and gene alignment analysis.** Contigs for the clade IV and clade I MAGs were reordered based on the *Synechococcus* reference genomes BL107 and CC9311 respectively using Mauve (version 2.4.0 (28). Annotations and gene locations for the reference genomes and the MAGs were produced as a gbk file by Prokka, and the annotated reordered MAGs were compared with the annotated reference genomes to identify whether the gaps for the missing nitrogen genes were located within or at the edge of the MAG contigs. GBK files of only the region in which the N genes are present for most *Synechococcus* species were generated by Prokka for the MAGs and the reference genomes BL107 (IV), CC9902 (IV), WH8016 (I), CC9311 (I), MIT S9220 (CRD1), and RS9917 (VIII). The nitrogen gene regions were then visualized and compared with Clinker (29). Nanopore long reads were mapped onto the MAGs with minimap2 and coverage of the nitrogen gene regions was visualized with IGV (21, 22).

**Nitrate reductase gene abundance and global data comparison.** *Synechococcus* nitrogen genes (nitrate and nitrite reductases and transporters, ammonia transporter, and urease subunit gamma in the EXPORTS samples were identified and quantified using keyword searches (‘*Synechococcus*’ to select for *Synechococcus* specific gene hits, and ‘nitrate reductase’, ‘nitrite reductase’, etc. to select the gene) within the diamond results of each EXPORTS sample. *Synechococcus* nitrate reductases were grouped by *Synechococcus* clade in order to compare their abundance to the *Synechococcus* MAG abundance and *Synechococcus* *recA* abundance.

For comparison of *Synechococcus* nitrate reductase gene frequencies between our sample and TARA samples, the nitrogen gene to *recA* ratio (nitrogen gene count per *Synechococcus* genome equivalent) was used for nitrate reductase *narB*, nitrite transporter *focA*, and the ammonia transporter *amt*. Raw forward and reverse metagenome reads (2x100 bp reads, 5 m depth, 0.2-3.0 um size fraction) from each TARA Station were collected from the European Nucleotides Archive (ENA) project PRJEB1787 and processed using the same workflow utilized to process the Station P dataset. In order to search through these large datasets, small databases containing all NCBI nitrate reductases, formate/nitrite transporters, and recombinase A sequences were used for the initial diamond blastx search to narrow down the query files to a smaller number of hits. The resulting read hits were then used as a query in a diamond blast against the entire NCBI Refseq v95 database to eliminate false hits, and for the *recA* sequences specifically, an additional diamond blast of these results was done against the GTDB RecA database to obtain GTDB taxonomic IDs for each *recA* hit. *Synechococcus* hits were then pulled out of the total bacterial results for each gene and quantified for calculation of the nitrogen gene to *recA* ratios, with only NCBI *Synechococcus* hits that maintained their *Synechococcus* taxonomic identification in GTDB being counted as true hits.

Iron and nitrate concentrations for each TARA station were calculated using the PISCES biogeochemistry model in order to relate nitrate reductase: *recA* to nitrate and iron availability at each station (30, 31).

**Data availability**. All metagenomes used for the assembly of the *Synechococcus* MAGs can be accessed under the NCBI Bioproject PRJNA78533 (Biosamples 24695624-24695646). These Whole Genome Shotgun projects have been deposited at DDBJ/ENA/GenBank under the accessions JAVBIW000000000 for *Synechococcus* sp. SP1 and JAVBIX000000000 for *Synechococcus* sp. SP2.


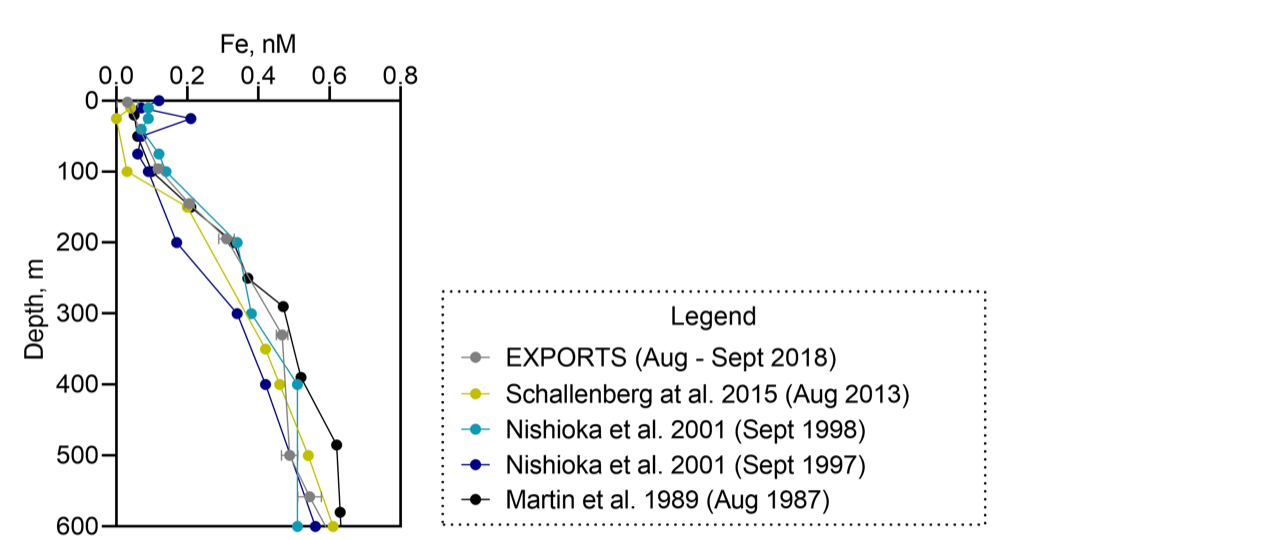


Figure S1. Averaged iron concentrations from 0-600 m in depth at Station Papa from the August-September 2018 EXPORTS cruise (gray), as well as from historic measurements from August 2013 (yellow), September 1998 (aqua), September 1997, (dark blue), and August 1987 (black).


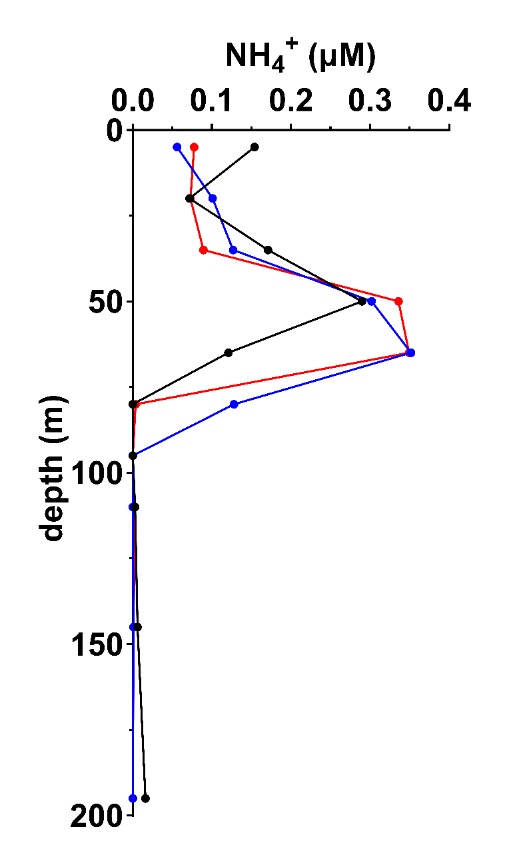

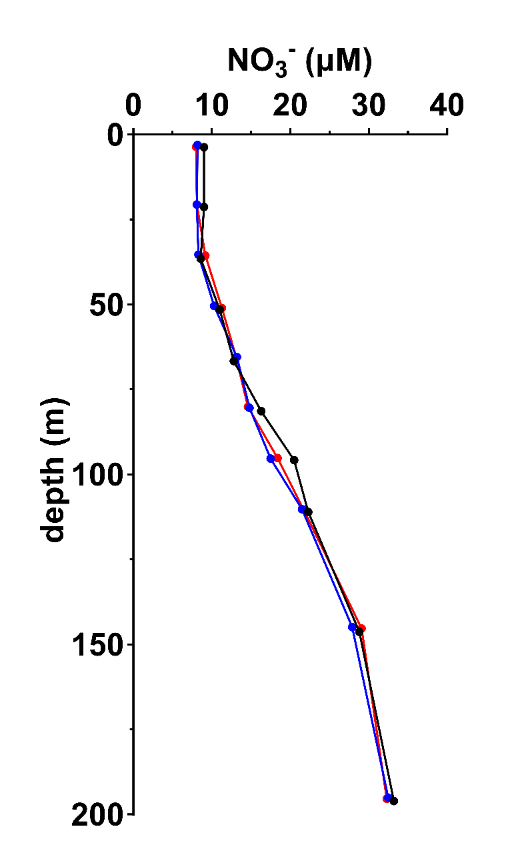

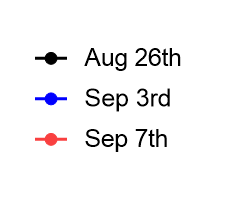


Figure S2. Ammonium and nitrate concentrations (µM) from 0-200 m in depth on August 26^th^ (black), September 3^rd^ (blue), and September 7^th^ (red).


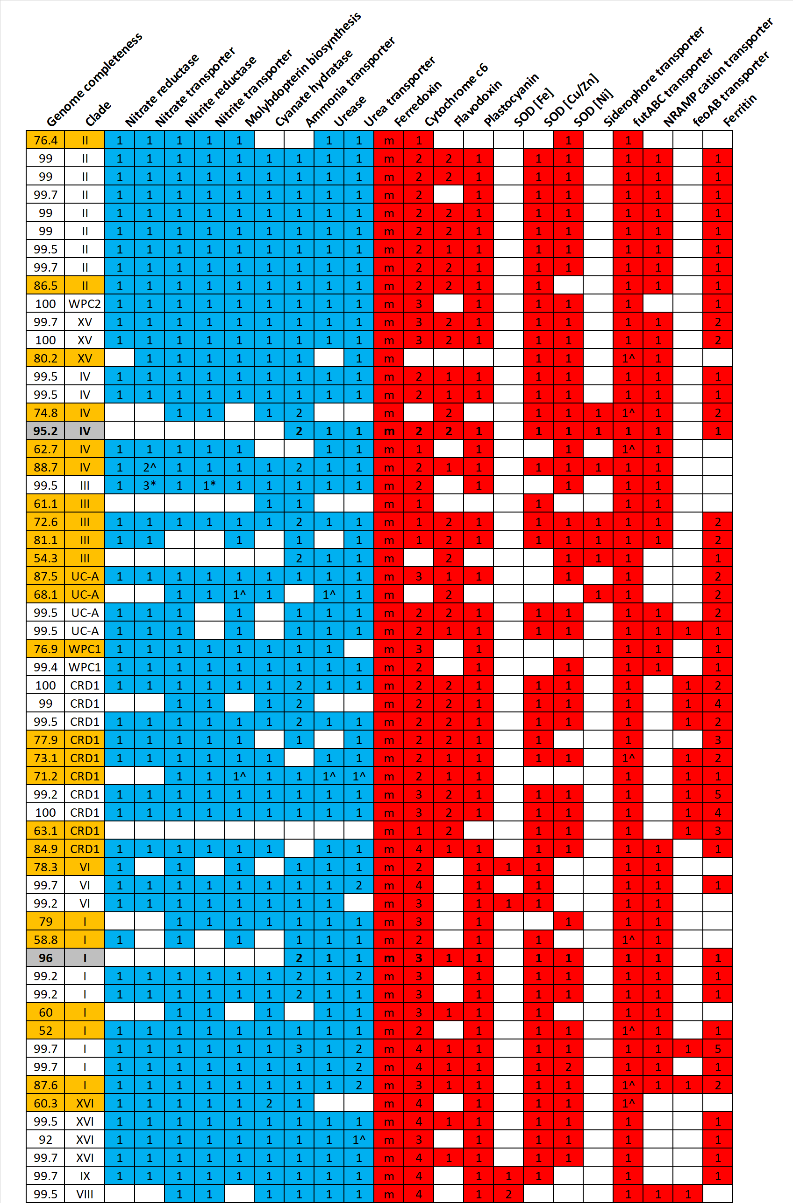

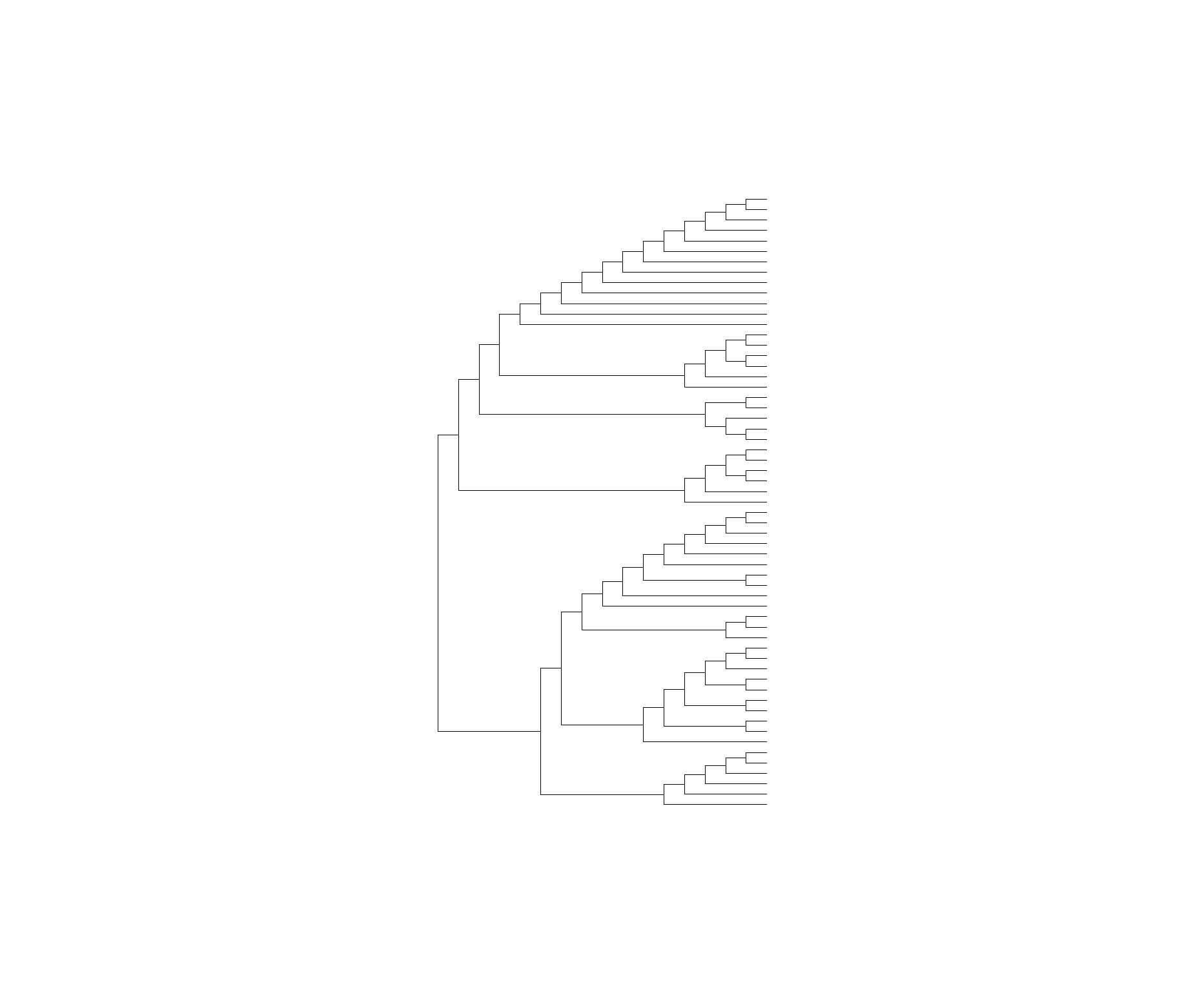


RS344

N5

N26

UW86

N19

N32

WH 8109

CC9605

AG-670-B23

KORDI-52

UW106

UW69

MED650

CC9902

BL107

AG-683-C23

**Syn SP1**

CPC35

ARS1019

WH 8102

TMED177

AG-679-D13

AG-679-B05

AG-673-B04

NAT70

AG-670-F04

CC9616

KORDI-100

MED850

KORDI-49

MIT S9508

MIT S9220

GEYO

AG-673-F03

AG-670-F22

AG-679-A04

MIT S9509

UW179A

ARS6

EAC657

TMED90

WH 7805

WH 7803

UBA7854

AG-686-A03

**Syn SP2**

WH 8016

UW179B

AG-686-F08

AG-683-A03

CC9311

WH8020

AG-679-C18

TMED187

UW105

NAT40

UW140

RS9916

RS9917

0.94

0.39

0.60

1.0

1.0

0.88

0.96

0.79

1.0

0.38

0.85

1.0

0.77

1.0

1.0

0.11

1.0

0.15

0.87

0.53

0.99

0.68

0.94

0.99

0.99

1.0

1.0

1.0

1.0

0.99

1.0

0.89

1.0

1.0

0.0

0.97

0.98

0.83

1.0

1.0

1.0

1.0

Figure S3. Phylogenomic distribution of nitrogen (blue) and iron (red) associated genes across all analyzed genomes of the *Synechococcus* clade. Station Papa *Synechococcus* MAGs are bolded and highlighted in gray. Numbers in a column represent gene copies in a genome, with m standing for multiple copies of the ferredoxin gene. Genomes with an estimate completeness of <90% are included here and highlighted in orange. * represent the inclusion of an active Nitrate/nitrite ABC transporter for WH 8102. ^ represent the presence of a partially complete pathway, where only some genes for that function were present within genomes with low completeness.


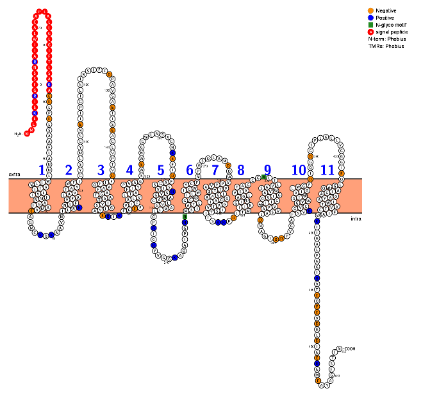


Extracellular charge:

With signal peptide -9

Without signal peptide -13

Intracellular charge +1


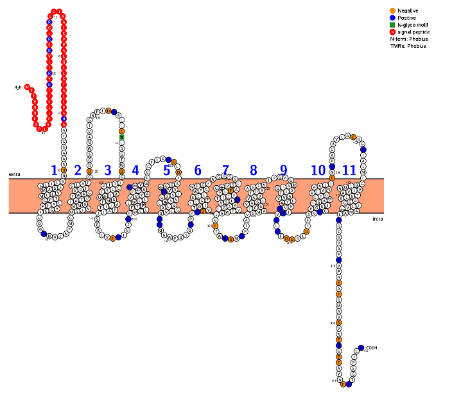


Extracellular charge:

With signal peptide +2

Without signal peptide -4

Intracellular charge +2

Syn SP1 transporters

Syn SP2 transporters


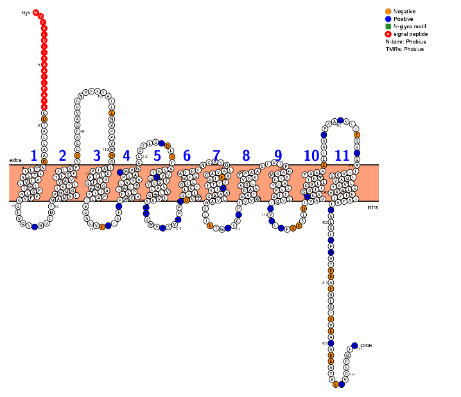


Extracellular charge:

With signal peptide -4

Without signal peptide -4

Intracellular charge +5

*A.*


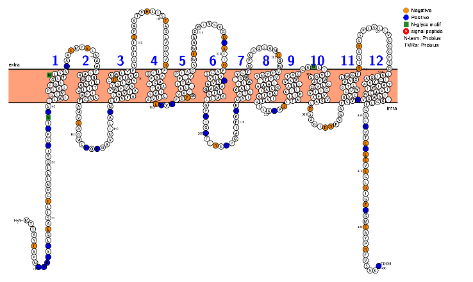


Extracellular charge:

With signal peptide -5

Without signal peptide -11

Intracellular charge -1

*B.*


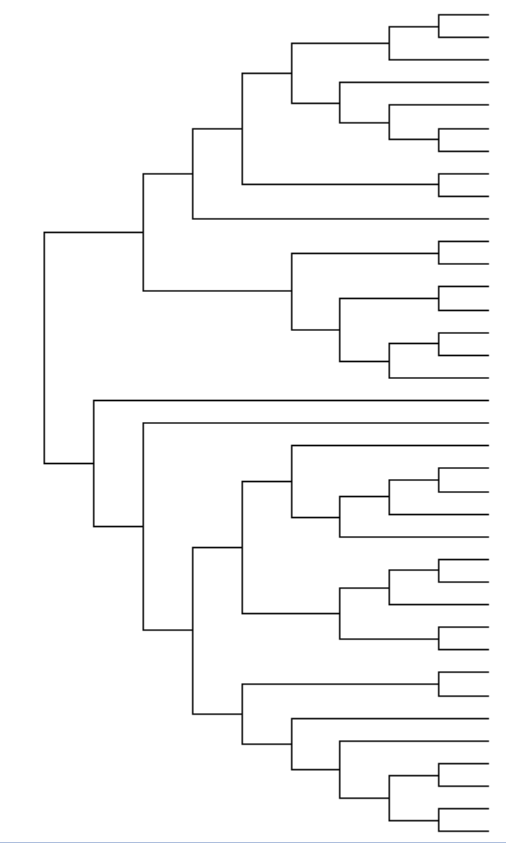


Synechococcus sp. CC9311_B

**Synechococcus sp. SP2_A**

0.9

0.75

1.0

0.79

0.84

0.82

0.98

0.98

0

0.79

0.83

0.68

1.0

0.78

0.87

1.0

0.9

0.97

0.88

0.71

0.97

Synechococcus sp. WH7803

Synechococcus sp. WH8016_B

**Synechococcus sp. SP1_A**

Synechococcus sp. CC9902

Synechococcus sp. BL107

Synechococcus sp. RS9917

Synechococcus sp. MITS9220_A

Prochlorococcus marinus strain NATL2A

Pelagibacter ubique strain HTCC1062

Alpha proteobaccterium HIMB114

Spiribacter salinus M19-40

Desulfonatronum thiosulfatophilum

Neptunomonas antarctica

Halomonas xianhensis

Endozoicomonas numazuensis

Nitrosopelagicus brevis CN25

Archaeoglobus fulgidus

Synechococcus sp. CC9311_A

Synechococcus sp. MITS9220_B

**Synechococcus sp. SP1_B**

**Synechococcus sp. SP2_B**

Synechococcus sp. WH8016_A

Methanosarcina acetivorans strain ATCC_35395_DSM_2834

Methanosarcina barkeri

Methanosarcina sp. MTP4

Kosmotoga olearia strain ATCC_BAA-1733_DSM21960

Kosmotoga pacifica

Escherichia coli strain K12

Nitrosopumilius maritimus SCM1

Oceanicoccus sagamiensis

Pelagibacter sp. Strain IMCC9063

Puniceispirillum marinum strain IMCC1322

SAR116 cluster alpha proteobacterium HIMB100

Roseobacter sp. SK209-2-6

Ruegeria lcuscaerulensis strain DSM_11314

0.93

0

0.85

0.98

1.0

0.87

0.28

0.47

0.98

0.22

0.89

0.78

0.94

1.0

Figure S4. A. Structure of Syn SP1 and SP2 ammonium transporters predicted using Protter  (32). Syn SP2 ammonium transporter A likely has a long signal motif that got mistaken as a 12^th^ transmembrane region. B. Phylogenetic tree   of ammonium transporters. Multiple sequence alignments of ammonia transporter amino acid sequences were performed with MUSCLE, and GBlocks removed poorly aligned and divergent regions of the sequence alignments and identified 174 positions to compare for phylogenetic analysis. PhyML generated the phylogenetic tree from the sequence alignments using an Approximate Likelihood-Ratio Test (aLRT) for branch support.


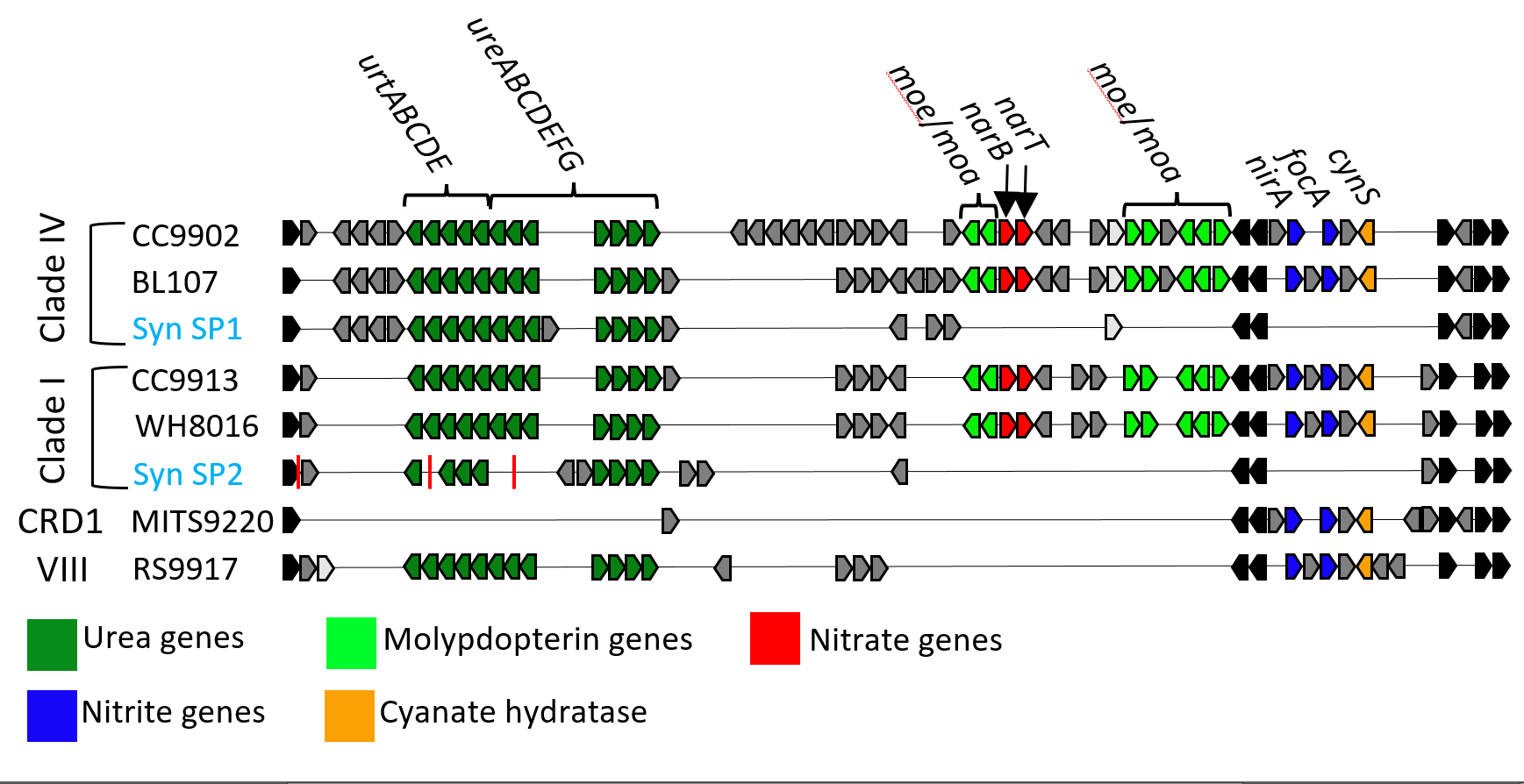

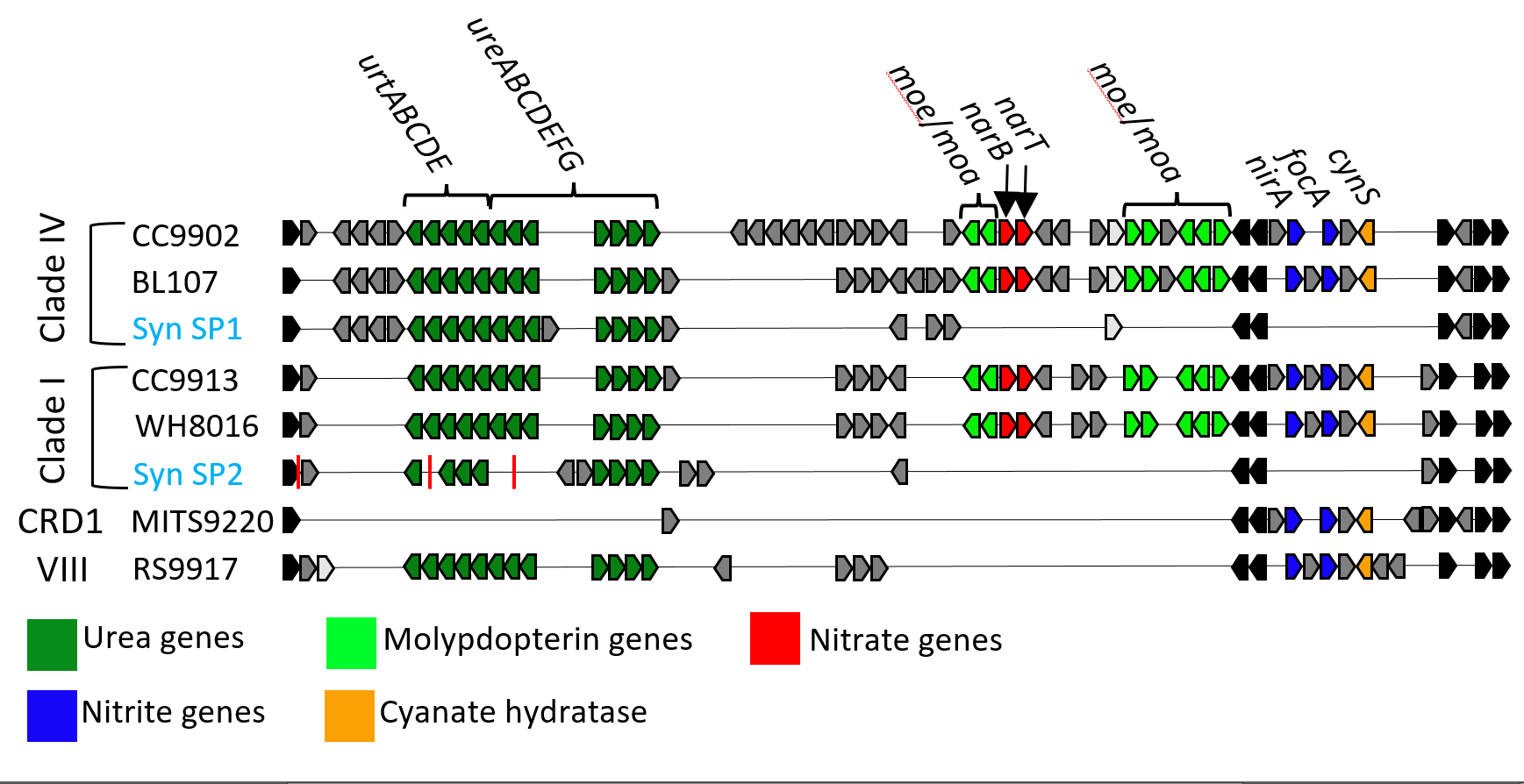

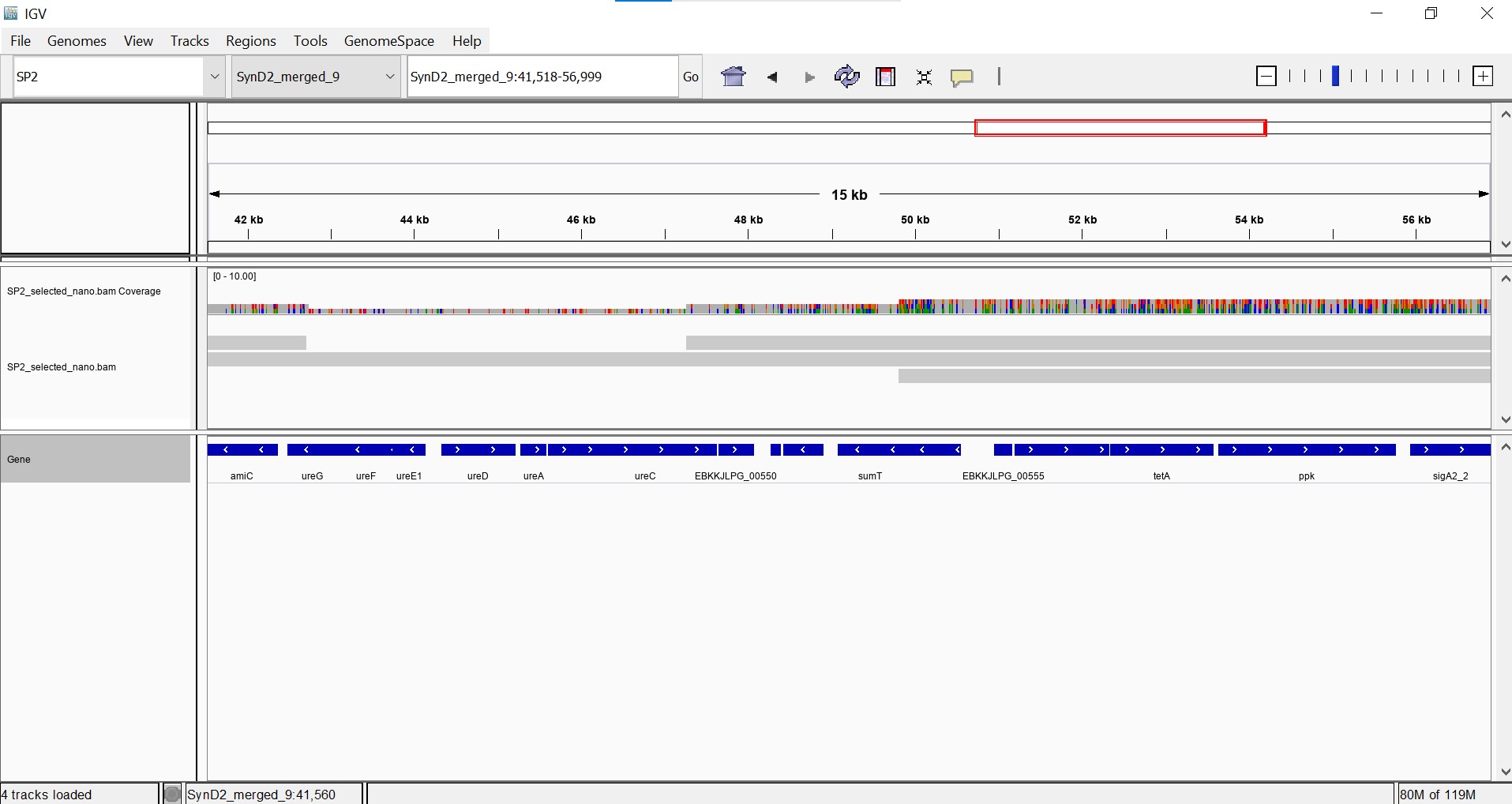

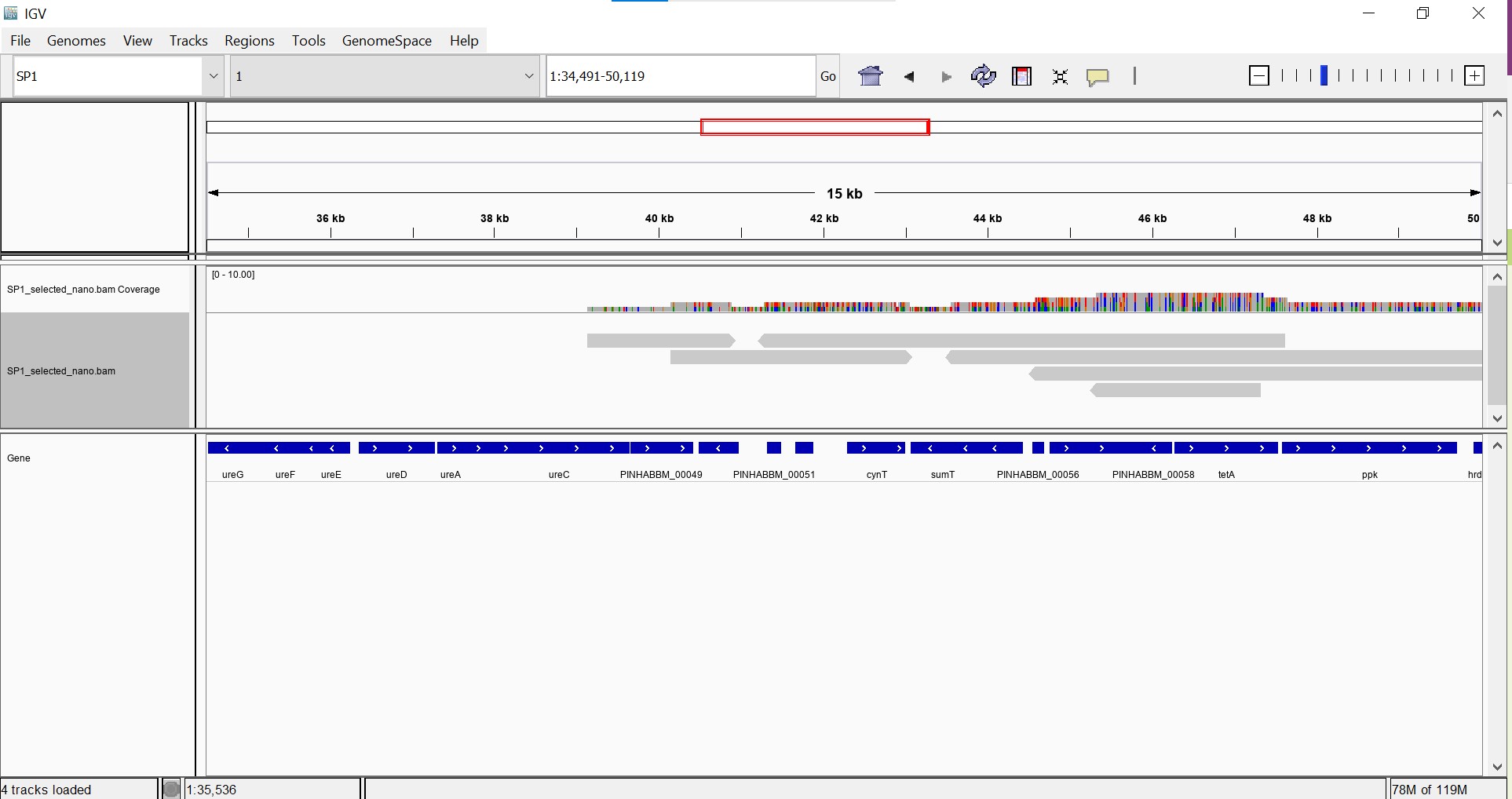


Synechococcus SP1 genome

Synechococcus SP2 genome

coverage

mapped long reads

CDS

CDS

coverage

mapped long reads

**narB**

**ureA**

**nirA**

**focA**

**narT**

**ureC**

44,058 bp

30,789 bp

26,575 bp

2,047 bp

21,217 bp

7,624 bp

2,900 bp

1,751 bp

7,113 bp

>

>

>

>

>

>

>

>

>

>

Figure S5. Nanopore long read coverage of the N gene region of the Synechococcus SP1 and SP2 MAGs. Arrows on the reads indicate that the read also maps to adjacent regions of the genome.

*B.*

*A.*


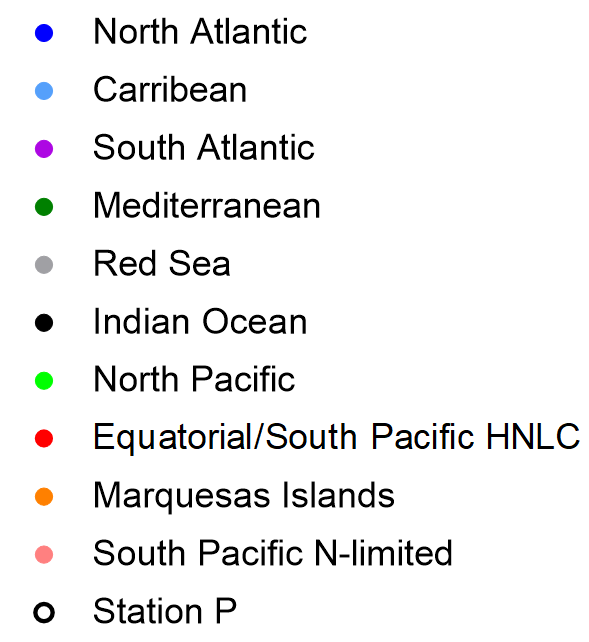


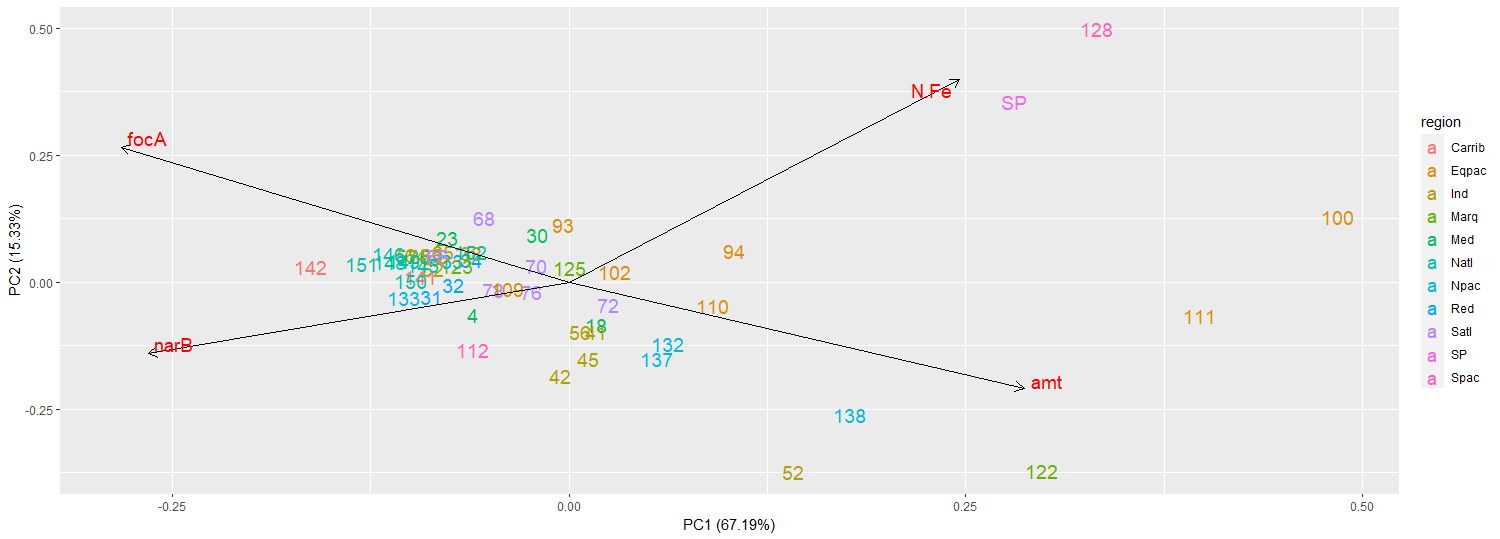


Figure S6. TARA analysis of various Synechococcus nitrogen genes. (A) Log_10_ normalized nitrate to iron ratio versus *Synechococcus* nitrite transporter (*focA*) and ammonium transporter (*amt*) gene copy number of the TARA stations and EXPORTS Station P. Select stations within the Equatorial Pacific HNLC zone and the Eastern Tropical Southern Pacific are labeled with their TARA ID. Red lines indicate line of best fit based on linear regression analysis (p-values not significant at 0.24 and 0.06 for *focA* and *amt* respectively). (B) Principal component analysis (PCA) of the TARA Stations, with narB copy number, *focA* copy number, *amt* copy number, and nitrogen to iron ratio as variables. Each station is labeled by their TARA station number (and SP for Station P) and a color corresponding to their respective ocean region, and loading arrows show how each variable (shown in red on graph) influences stations on the PCA plot.

-80

-60

-40

-20

0

20

Latitude

40

60

80

-150

-100

-50

0

50

100

0

15

25

35

30

20

10

5

Annual Nitrate at 5m (µmol kg^-1^)


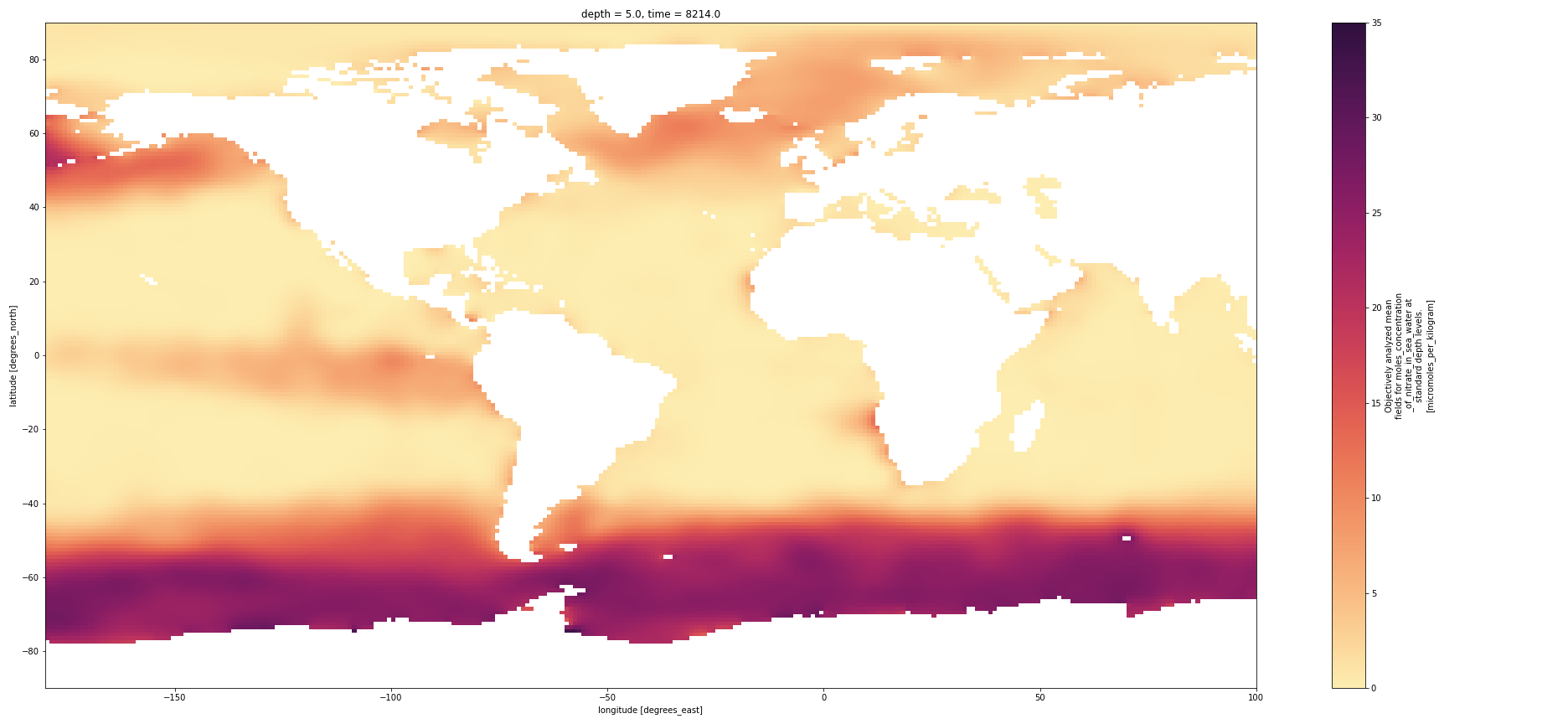


Station P

Metzyme Stations

1

5

ETSP Stations

1

3

5


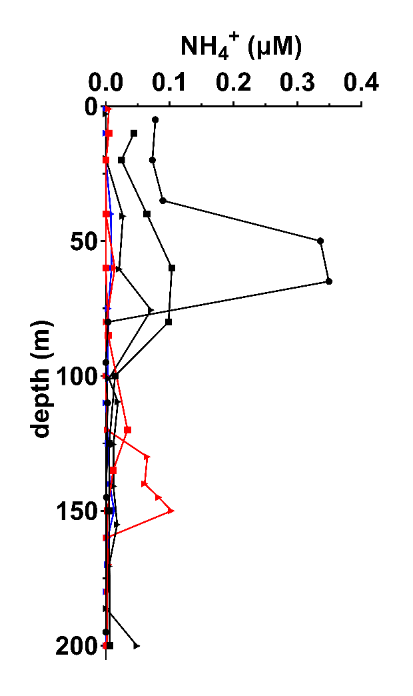

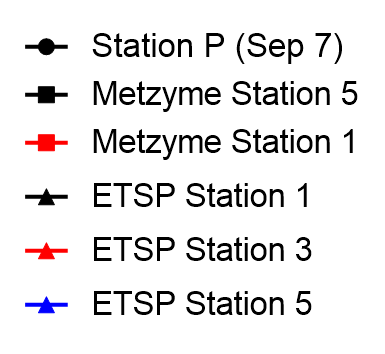


Longitude

Figure S7. Comparison of ammonium concentration depth profiles between the Northeastern Pacific (Station P September 7^th^), the Central Equatorial Pacific, and the Southeastern Pacific. The Metzyme cruise stations are located at the equator (Station 1, 0 degrees latitude) and in the Northern Tropical Pacific (Station 5, 8 degrees latitude) (data from BCO-DMO database Project#2236 Dataset#646115) (33). Eastern Tropical South Pacific Stations (ETSP) are all located on a horizontal transect at -20 degrees latitude (-70, -80, and -100 degrees longitude for Stations 1, 3, and 5 respectively) (data from BCO-DMO database Project#555516 Dataset#820165) (34).

**Citations**

1. Siegel DA, Cetinić I, Graff JR, Lee CM, Nelson N, Perry MJ, et al. An operational overview of the EXport Processes in the Ocean from RemoTe Sensing (EXPORTS) Northeast Pacific field deployment [Internet]. Vol. 9, Elem Sci Anth. University of California Press; 2021. p. 00107.
2. Measures CI, Landing WM, Brown MT, Buck CS. A commercially available rosette system for trace metal-clean sampling. *Limnol Oceanogr Methods* 2008; **6**: 384–394.
3. Burns SM, Bundy RM, Abbott W, Abdala Z, Sterling AR, Chappell PD, et al. Interactions of bioactive trace metals in shipboard Southern Ocean incubation experiments. *Limnol Oceanogr* 2023.
4. Burns SM, Buck K, Jenkins BD, Brzezinski MA. Dissolved trace metal (Mn, Fe, Co, Ni, Cu, Zn, Cd, Pb) concentration data from surface (towfish) samples collected during 2018 EXPORTS North Pacific cruise to the subarctic North Pacific near Ocean Station PAPA (Station P) on R/V Roger Revelle RR1813. Biological and Chemical Oceanography Data Management Office (BCO-DMO) 2022. (Version 1) Version Date 2022-03-10. doi:10.26008/1912/bco-dmo.869683.1
5. Graff JR, Behrenfeld MJ. Photoacclimation Responses in Subarctic Atlantic Phytoplankton Following a Natural Mixing-Restratification Event. Frontiers in Marine Science [Internet]. 2018;5.
6. Andrews S, Others. FastQC: a quality control tool for high throughput sequence data. Babraham Bioinformatics, Babraham Institute, Cambridge, United Kingdom; 2010.
7. Bolger AM, Lohse M, Usadel B. Trimmomatic: a flexible trimmer for Illumina sequence data. Bioinformatics. 2014 Aug 1;30(15):2114–20.
8. Zhang J, Kobert K, Flouri T, Stamatakis A. PEAR: a fast and accurate Illumina Paired-End reAd mergeR. Bioinformatics. 2014 Mar 1;30(5):614–20.
9. Buchfink B, Xie C, Huson DH. Fast and sensitive protein alignment using DIAMOND. Nat Methods. 2015 Jan;12(1):59–60.
10. De Coster W, D’Hert S, Schultz DT, Cruts M, Van Broeckhoven C. NanoPack: visualizing and processing long-read sequencing data. Bioinformatics. 2018 Aug 1;34(15):2666–9.
11. Nurk S, Meleshko D, Korobeynikov A, Pevzner PA. metaSPAdes: a new versatile metagenomic assembler. Genome Res. 2017 May;27(5):824–34.
12. Langmead B, Salzberg SL. Fast gapped-read alignment with Bowtie 2. Nat Methods. 2012 Mar 4;9(4):357–9.
13. Li H, Handsaker B, Wysoker A, Fennell T, Ruan J, Homer N, et al. The Sequence Alignment/Map format and SAMtools. Bioinformatics. 2009 Aug 15;25(16):2078–9.
14. Kang DD, Li F, Kirton E, Thomas A, Egan R, An H, et al. MetaBAT 2: an adaptive binning algorithm for robust and efficient genome reconstruction from metagenome assemblies. PeerJ. 2019 Jul 26;7:e7359.
15. Wu Y-W, Tang Y-H, Tringe SG, Simmons BA, Singer SW. MaxBin: an automated binning method to recover individual genomes from metagenomes using an expectation-maximization algorithm. Microbiome. 2014 Aug 1;2:26.
16. Alneberg J, Bjarnason BS, de Bruijn I, Schirmer M, Quick J, Ijaz UZ, et al. CONCOCT: Clustering cONtigs on COverage and ComposiTion [Internet]. arXiv [q-bio.GN]. 2013. Available from: <http://arxiv.org/abs/1312.4038>
17. Sieber CMK, Probst AJ, Sharrar A, Thomas BC, Hess M, Tringe SG, et al. Recovery of genomes from metagenomes via a dereplication, aggregation and scoring strategy. Nat Microbiol. 2018 Jul;3(7):836–43.
18. Parks DH, Imelfort M, Skennerton CT, Hugenholtz P, Tyson GW. CheckM: assessing the quality of microbial genomes recovered from isolates, single cells, and metagenomes. Genome Res. 2015 Jul;25(7):1043–55.
19. Chaumeil P-A, Mussig AJ, Hugenholtz P, Parks DH. GTDB-Tk: a toolkit to classify genomes with the Genome Taxonomy Database. Bioinformatics [Internet]. 2019 Nov 15; Available from: <http://dx.doi.org/10.1093/bioinformatics/btz848>
20. Treangen TJ, Sommer DD, Angly FE, Koren S, Pop M. Next generation sequence assembly with AMOS. Curr Protoc Bioinformatics. 2011 Mar;Chapter 11(1):Unit 11.8.
21. Li H. Minimap2: pairwise alignment for nucleotide sequences. Bioinformatics. 2018 Sep 15;34(18):3094–100.
22. Robinson JT, Thorvaldsdóttir H, Winckler W, Guttman M, Lander ES, Getz G, et al. Integrative genomics viewer. Nat Biotechnol. 2011 Jan;29(1):24–6.
23. Eren AM, Esen ÖC, Quince C, Vineis JH, Morrison HG, Sogin ML, et al. Anvi’o: an advanced analysis and visualization platform for 'omics data. PeerJ. 2015 Oct 8;3:e1319.
24. Hyatt D, Chen G-L, Locascio PF, Land ML, Larimer FW, Hauser LJ. Prodigal: prokaryotic gene recognition and translation initiation site identification. BMC Bioinformatics. 2010 Mar 8;11:119.
25. Parks DH, Chuvochina M, Chaumeil P-A, Rinke C, Mussig AJ, Hugenholtz P. A complete domain-to-species taxonomy for Bacteria and Archaea. Nat Biotechnol. 2020 Sep;38(9):1079–86.
26. Satinsky BM, Gifford SM, Crump BC, Moran MA. Use of internal standards for quantitative metatranscriptome and metagenome analysis. Methods Enzymol. 2013;531:237–50.
27. Gifford SM, Zhao L, Stemple B, DeLong K, Medeiros PM, Seim H, et al. Microbial Niche Diversification in the Galápagos Archipelago and Its Response to El Niño. Front Microbiol. 2020 Oct 23;11:575194.
28. Darling ACE, Mau B, Blattner FR, Perna NT. Mauve: multiple alignment of conserved genomic sequence with rearrangements. Genome Res. 2004 Jul;14(7):1394–403.
29. Gilchrist CLM, Chooi Y-H. Clinker & clustermap.js: Automatic generation of gene cluster comparison figures. Bioinformatics [Internet]. 2021 Jan 18; Available from: <http://dx.doi.org/10.1093/bioinformatics/btab007>
30. Aumont O, Ethé C, Tagliabue A, Bopp L, Gehlen M. PISCES-v2: an ocean biogeochemical model for carbon and ecosystem studies. Geosci Model Dev. 2015 Aug 13;8(8):2465–513.
31. Richon C, Tagliabue A. Biogeochemical feedbacks associated with the response of micronutrient recycling by zooplankton to climate change. Glob Chang Biol. 2021 Oct;27(19):4758–70.
32. Omasits U, Ahrens CH, Müller S, Wollscheid B. Protter: interactive protein feature visualization and integration with experimental proteomic data. Bioinformatics. 2014 Mar 15;30(6):884–6.
33. Saito MA. Nutrients, targeted proteomics, and pigments from the METZYME cruise (KM1128). Biological and Chemical Oceanography Data Management Office (BCO-DMO) Version Date 2018-05-24 (2018).
34. Casciotti KL, Santoro AE, Knapp AN, Dissolved nitrite and ammonium concentration data from R/V Atlantis (AT15-61) cruise in Jan-Feb 2010 and R/V Melville (MV1104 cruise in Mar-Apr 2011 in the Eastern Tropical South Pacific. Biological and Chemical Oceanography Data Management Office (BCO-DMO) Version Date 2020-08-06 (2021).
